# Assimilation of high-resolution Ocean Color Monitor (OCM) aerosol optical depth in WRF-Chem improves PM₂.₅ forecasts over the Indian region

**DOI:** 10.1038/s41598-025-23307-1

**Published:** 2025-11-12

**Authors:** Prafull P. Yadav, Sachin D. Ghude, Rajesh Kumar, Gaurav Govardhan, Rajmal Jat, Shivani Shah, B. P. Shukla, Manoj K. Mishra, Deepak Putrevu, P. K. Thapliyal, Rashmi Sharma

**Affiliations:** 1 Indian Institute of Tropical Meteorology, Ministry of Earth Sciences, Pune, India; 2 Department of Atmospheric and Space Sciences, Savitribai Phule Pune University, Pune, India; 3 National Center for Medium Range Weather Forecasting, Ministry of Earth Sciences, Noida, Uttar Pradesh India; 4 National Science Foundation National Center for Atmospheric Research, Boulder, CO USA; 5 Space Applications Centre, Ahmedabad, India

**Keywords:** Ocean Color Monitor (OCM), WRF-Chem, Satellite data assimilation, Atmospheric chemistry, Pollution remediation, Scientific data, Astronomical instrumentation

## Abstract

This study investigates the impact of assimilating high resolution (770 m) Aerosol Optical Depth (AOD) retrieval derived from the Oceansat-3 Ocean Colour Monitor (OCM) sensor into the Weather Research and Forecasting model coupled with Chemistry (WRF-Chem) for the first time, aiming to improve fine particulate matter (PM₂.₅) forecasts over India. AOD assimilation, evaluated over the period 01–15 November 2023, leads to substantial improvements in model accuracy, reducing PM₂.₅ biases by 30–70% and lowering root mean square error (RMSE) across critical regions such as Delhi, Punjab, Bihar, and West Bengal. The assimilation substantially improves initial conditions of surface PM₂.₅ estimates by approximately 60 µg/m³. Forecast accuracy is the highest on the first day, with an RMSE of 21.35 µg/m³ and a correlation coefficient (R) of 0.75, followed by increasing RMSE values of 30.40 µg/m³ on Day 2 and 32 µg/m³ on Day 3, with correlations of 0.73 and 0.70, respectively, reflecting degradation of assimilation benefits by model uncertainties over time. With MODIS nearing phase-out, high-resolutionOCM AOD retrievals offer a reliable alternate choice for future AOD assimilation in the AIRWISE forecasting system over India.

**Supplementary Information:**

The online version contains supplementary material available at 10.1038/s41598-025-23307-1.

## Introduction

India’s rapid urbanization, industrialization, and rising energy demand have driven a sharp increase in anthropogenic emissions, worsening air quality nationwide^1,2^. Widespread PM₂.₅ pollution—linked to vehicular, industrial, and agricultural sources—is often exacerbated by unfavourable meteorology^3–5^ posing serious environmental and public health risks. Accurate short-term forecasts are therefore critical, enabling timely advisories and interventions that reduce exposure during high-pollution episodes, particularly for vulnerable groups^6,7^. A reliable air quality forecasting is essential for Indian cities due to the severe and persistent air pollution challenges driven by rapid urbanization, industrialization, and vehicular emissions. With frequent episodes of high pollution levels, accurate forecasting helps provide timely Air Quality Index (AQI) information to the public, enabling precautionary measures to reduce health risks, especially for vulnerable populations^8^.

To address this challenge, the Ministry of Earth Sciences (MoES) developed the Air Quality Early Warning System (AQEWS) for the National Capital Region (NCR)^9–11^. Over time, AQEWS evolved into the Air Quality Warning and Integrated Decision Support System for Emissions (AIRWISE)^12,13^, a cutting-edge tool that combines high-resolution numerical modeling based air quality forecasts with actionable source attribution insights for air quality management. This system helps policymakers to implement the Graded Response Action Plan (GRAP) in Delhi-NCR, which imposes temporary predefined restrictions on pollution sources based on forecast data. The integrated system helps in managing air quality during critical periods like Diwali (a major Indian festival celebrated with fireworks and lights) and other high pollution events, providing crucial support for public health and environmental management. AIRWISE employs advanced chemical-transport models (e.g., WRF-Chem) within a three-dimensional variational (3DVAR) data assimilation framework, integrating ground-based measurements with satellite-derived Aerosol Optical Depth (AOD) products—particularly from the Moderate Resolution Imaging Spectroradiometer (MODIS). Assimilation of MODIS AOD has substantially improved PM₂.₅ forecasts^14^, however, with the MODIS platform nearing the end of its operational lifespan^15^ the need to explore alternative satellite systems has become increasingly urgent to ensure continuity and advancement in data assimilation efforts. In this context, Oceansat-3 Ocean Colour Monitor (OCM) onboard the Indian Space Research Organization’s (ISRO) satellite EOS-06, launched in 2022, presents a promising alternative. Although primarily designed for oceanographic applications, OCM extends its capabilities to aerosol monitoring over land, offering enhanced spatial and temporal resolution for air quality modeling. The transition from MODIS to OCM provides an opportunity to maintain and improve the accuracy of AIRWISE by ensuring the consistency of AOD retrievals in the data assimilation pipeline. This integration of OCM AOD retrievals into AIRWISE forms the core of the present study. By leveraging the advanced features of OCM, the research aims to refine PM_2.5_ predictions during peak pollution periods and support evidence-based decision-making to mitigate air quality crises. The findings are expected to contribute to the ongoing evolution of air quality forecasting systems, emphasizing the critical role of satellite-based data in tackling complex environmental challenges in India and beyond.

In this paper, we highlight the impact of OCM data assimilation on air quality forecasting and assess how it performs during peak pollution periods compared to MODIS. Our analysis shows that the OCM-assimilated forecast falls within the expected uncertainties and is reliable for issuing timely warnings, demonstrating its suitability alongside MODIS for operational air quality forecasting.

## Data and methods

### Satellite retrievals for data assimilation

This research utilizes the WRF-Chem model configuration, emissions, parameterization schemes as employed in the AIRWISE framework^9,13^ and also provided in the supplementary document under the section “WRF-Chem Model Configuration”, focusing with 10 km grid spacing over the entire Indian region as the domain of analysis. For context, the spatial pattern of open-biomass-burning PM₂.₅ emissions used in the model (FINN) during the study period is shown in Supplementary Fig. S2 (PM₂.₅ emission flux from fires, averaged over 13 days).The meteorological initial and boundary conditions were derived from the analysis and forecast product of the Indian Institute of Tropical Meteorology-Global Forecasting System (IITM-GFS, T1534). This system employs Ensemble Kalman Filtering at a horizontal resolution of 12.5 km, providing data every three hours^16^.

We have performed three experiments. The first was a control run without data assimilation, referred to as the CNTL experiment; the second included assimilation using MODIS AOD data, defined as the MODISDA experiment; and the third incorporated assimilation using OCM AOD data, termed the OCMDA experiment. All simulations were performed with daily forecast outputs generated for 72 h. The meteorological parameters are reinitialized using global weather forecasts from IITM-GFS and chemistry was recycled from the previous day’s forecast to generate background initial conditions at the start of each forecast cycle. The analysis period spanned from 01 November 2023 to 14 November 2023, selected to ensure high AOD data availability with minimal persistent cloud cover during. To ensure consistency and comparability, all parameterization schemes and model configurations were kept identical across the three experiments.

To assimilate either MODIS or OCM AOD in WRF-Chem, the 3DVAR data assimilation framework within the Gridpoint Statistical Interpolation (GSI) system is utilized. AOD assimilation leverages the observational data to optimize model initial conditions of aerosol species by minimizing a cost function J, expressed as:

1 $$\:J\left(x\right)\:=\:\frac{1}{2}{(x\:-\:{x}_{b})}^{T}{B}^{-1}(x\:-\:{x}_{b})\:+\:\frac{1}{2}{\left(H\right(x)\:-\:y)}^{T}{R}^{-1}\left(H\right(x)\:-\:y)$$

Here, *x* is the state vector representing model variables (aerosol species and meteorological parameters required for AOD calculation), *x*_*b*_ is the background model state, B is the background error covariance matrix, H is the forward operator transforming model variables (aerosols) to observational (AOD) space, *y* represents the satellite AOD observations, and *R* is the observation error covariance matrix. The forward operator employs the Community Radiative Transfer Model (CRTM) to compute AOD from modeled aerosol concentrations. The assimilation process iteratively minimizes the cost function by balancing the model background with observations, resulting in updated aerosol mass concentrations and improved analysis accuracy for initialization of air quality predictions. The background error covariance matrix is estimated using the National Meteorological Center (NMC) method through the Generalized Background Error (GEN_BE) module. This approach uses differences between two forecasts valid at the same time to estimate parameters such as variance, horizontal length scales, and vertical length scales. These parameters are derived from daily pairs of 24-hour WRF-Chem forecasts initialized under varying meteorological, anthropogenic, and biomass burning conditions, We consider a 100% uncertainty in both anthropogenic and biomass burning emissions, based on intercomparison studies of various emission inventories^18,19,17^. The variances determine the weight of observational innovations, while the length scales govern the spatial and vertical influence of assimilation increments^10^. Further technical details of the GSI 3DVAR configuration are provided in Supplementary Section S3.

The MODIS instrument, onboard NASA’s Terra and Aqua satellites launched in 1999 and 2002, respectively, operates in a sun-synchronous orbit with equatorial overpass times at approximately 10:30 AM and 1:30 PM local time. It provides near-global coverage every 1–2 days with a wide swath width of approximately 2330 km. It offers AOD retrievals at spatial resolutions of 10 km, derived using the Dark Target (DT) and Deep Blue (DB) algorithms. MODIS retrievals have observation errors estimated as ±(0.05 + 0.15 × AOD) over land and ±(0.03 + 0.05 × AOD) over ocean. In this study, MODISDA benefits from two daily overpasses (Terra and Aqua), which increases the probability of obtaining cloud-free retrievals for assimilation. Spectrally, MODIS has 36 bands spanning ~ 0.41–14.4 μm, covering the visible and near-infrared (VNIR; 0.40–1.0 μm), shortwave infrared (SWIR; 1.0–2.5 μm), and thermal infrared (TIR; 8–14 μm) ranges; the Dark Target/Deep Blue AOD retrievals mainly use visible blue/green/red bands (≈ 0.47–0.67 μm) together with the 2.13 μm SWIR band over land (and multiple VNIR bands over ocean). No additional bias correction was applied to either OCM or MODIS AOD prior to assimilation. The assimilation used the standard retrievals from the respective algorithms (OCM: SAER; MODIS: Collection 6.1 merged Dark Target/Deep Blue) without region-specific adjustment. Differences in surface reflectance can introduce systematic biases in AOD, particularly over bright or heterogeneous surfaces, which may influence DA performance and contribute to some of the observed regional variations.

In line with MODIS, the OCM, onboard ISRO’s Oceansat-3 satellite launched in 2022, also operates in a sun-synchronous orbit with an equatorial overpass time near 12:00 PM local time. OCM has a narrower swath width of approximately 1420 km, providing high-resolution AOD retrievals at a spatial resolution of approximately 770 m (0.007°), making it particularly suited for aerosol monitoring over South Asia and adjoining regions. OCM retrievals, produced using the Space Applications Center AErosol Retrieval (SAER) algorithm, have a theoretical uncertainty of ±(0.06 + 0.26 × AOD) over land^19^. Unlike MODIS, OCM provides only a single daytime overpass and, in our configuration, data were available on alternate days, making OCMDA more sensitive to cloud cover coinciding with the overpass. The study period was chosen to minimise persistent cloud cover so that OCM retrievals were available for most regions and days. OCM provides 13 visible–near-infrared (VNIR; 0.40–1.01 μm) bands (412, 443, 490, 510, 555/566, 620, 670/681, 710, 780, 870, 1010 nm); we assimilate the 870-nm AOD. OCM lacks mid-infrared (MIR; 3–5 μm) channels, so it cannot perform MIR-based active-fire detection. These differences in overpass frequency, spectral coverage, and cloud sensitivity directly affect the number of assimilated observations (see Supplementary Table S3).

MODIS near real-time retrievals have a latency of approximately 3 h, while OCM retrievals are available with a shorter latency of 2 h, making both datasets accessible by around 16:30 IST every day. In the operational forecasting setup, downloading and processing these near real-time AOD retrievals takes about 15 min. Each day, the chemical fields are initialized from the previous day’s WRF-Chem forecast, aerosol fields are updated through data assimilation, and meteorological fields are refreshed using the IITM-GFS forecast. This workflow ensures that applying assimilation at 09 UTC is both practical and efficient. PM₂.₅ measurements from 258 monitoring sites, located in urban centers across India, are collected by the Central Pollution Control Board (CPCB), IITM, and the US Embassies’ AirNow program. These sites are distributed across 12 states: 40 sites in Delhi, 17 in Gujarat, 30 in Haryana, 2 in Jharkhand, 1 in West Bengal (Kolkata US Embassies site), 29 in Madhya Pradesh, 11 in Pune (Maharashtra, IITM sites), 19 in Odisha, 8 in Punjab, 44 in Rajasthan, and 57 in Uttar Pradesh. These locations were judiciously chosen as MODIS and OCM satellites have daily swath coverage over these regions during the study period. The geographical locations of these 12 states are shown in Fig. S1 and were used to evaluate the performance of the assimilation experiments. However, as the network is uneven and urban-biased, unmonitored regions are treated as less directly validated, even though satellite AOD assimilation provides broader spatial constraints. To ensure the reliability of PM_2.5_ observations for evaluating model performance, additional quality control steps were applied alongside the standard procedures implemented by the CPCB (https://cpcb.nic.in/quality-assurance-quality-control/). First, measurements below 10 µg m^− 3^ and above 1,500 µg m^− 3^ were excluded as such extreme values were likely due to instrument malfunctions. For example, PM_2.5_ concentrations below 10 µg m^− 3^ were often recorded immediately after instrument restarts, which is improbable in regions with significant anthropogenic emissions. Second, sporadically high PM_2.5_ values, which appeared intermittently in the time series at some monitoring sites, were also filtered out.

A variety of statistical metrics were employed to evaluate the performance of the model experiments, including Pearson’s correlation coefficient (R), mean bias (MB), and root mean square error (RMSE). Pearson’s correlation coefficient was interpreted using its statistical significance to ensure robust insights into the relationships between observed and forecasted values. These metrics comprehensively assess the accuracy and reliability of day-1 to day-3 PM_2.5_ forecasts, offering valuable insights into the model’s performance.

## Results

### Effect of aerosol optical depth assimilation on PM_2.5_ forecast

**Fig. 1:**
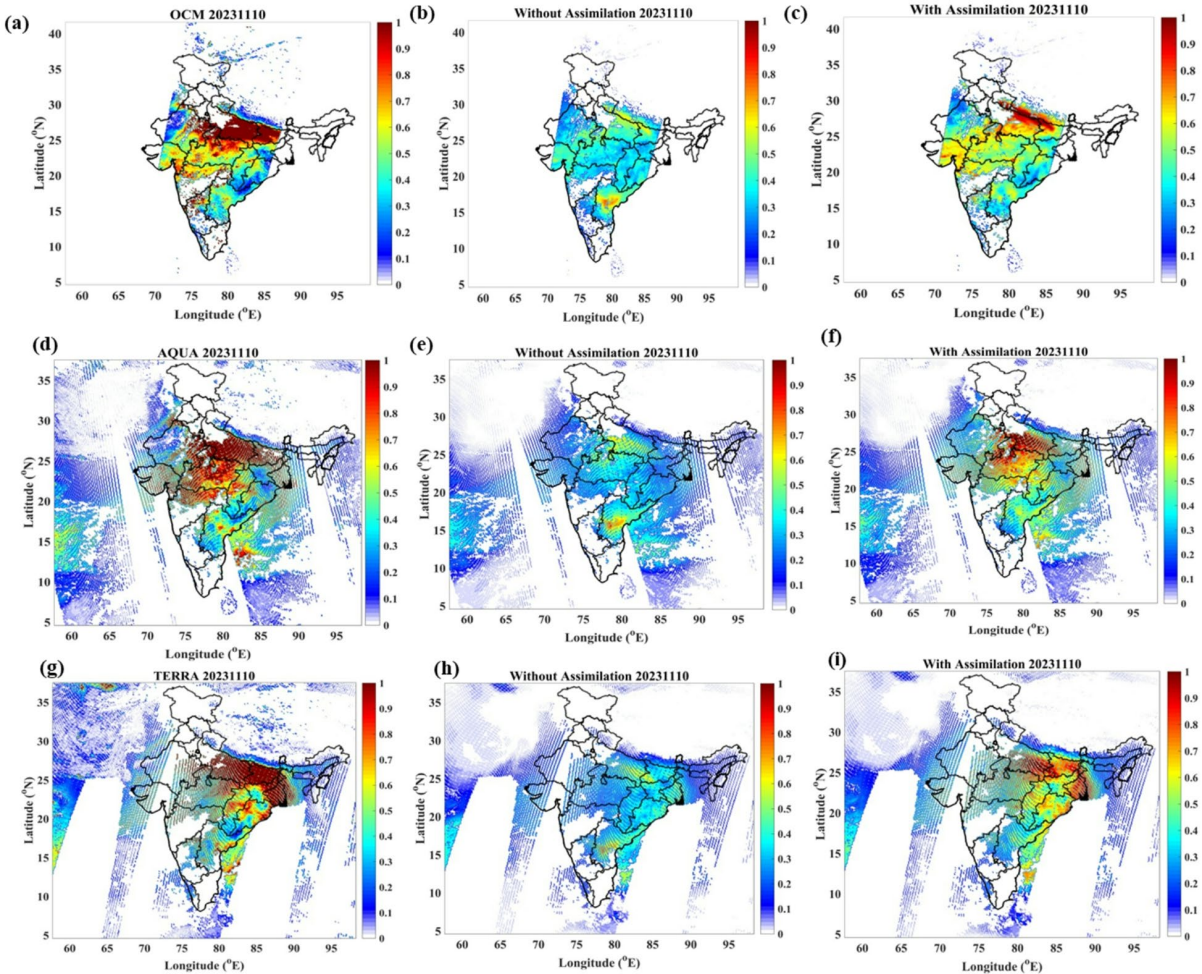
Spatial distribution of AOD at 550 nm on 10 November 2023 at 09:00 UTC, comparing observations and model simulations. The first column shows observed AOD from OCM, Aqua MODIS, and Terra MODIS satellite swaths (panels **a, d, g**). The second column presents WRF-Chem simulations without data assimilation (CNTL experiment; panels **b, e, h**).The third column displays simulations after assimilating satellite AOD: OCM AOD assimilation (OCMDA; panel **c**) and MODIS AOD assimilation (MODISDA; panels **f, i**).

**Fig. 2:**
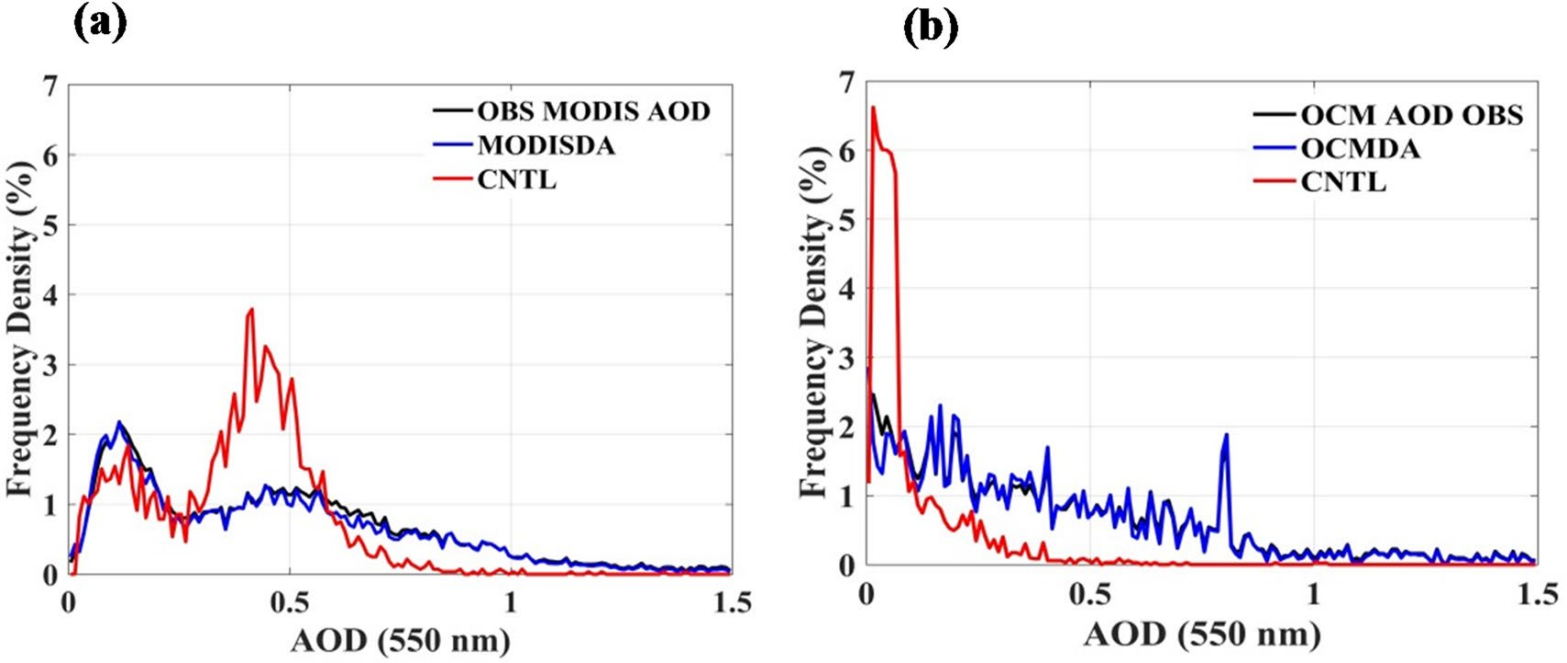
Frequency distribution of AOD from observations and model simulations during 1–14 November 2023 at 09:00 UTC. (**a**) Comparison of WRF-Chem simulations without assimilation (CNTL) and with MODIS AOD assimilation (MODISDA) against collocated MODIS observations. (**b**) Comparison of CNTL and OCM AOD assimilation (OCMDA) against collocated OCM observations.

To evaluate the effectiveness of OCMDA in improving WRF-Chem simulated AOD and to understand how chemical data assimilation enhances aerosol initial conditions, we have compared the CNTL and OCMDA experiments against collocated OCM AOD retrievals on 10 November 2023 at 09 UTC (Fig. 1). OCM provides substantially finer spatial sampling (~ 770 m), which has been shown in earlier studies (Mishra et al., 2023^19^ and Khan et al., 2025^20^) to capture localized aerosol features — such as narrow plumes and emission hotspots that are critical for detailed regional air-quality assessment. In this study, we first assessed the product at 10 km resolution to establish robustness over a large domain, with higher‐resolution analyses planned for future work. OCM provides higher spatial resolution data, which can be critical for detailed regional studies that require finer spatial granularity. The CNTL experiment (Fig. 1b) underestimates AOD substantially when compared with OCM observations (Fig. 1a), with simulated AOD values ranging between 0.1 and 0.4, while observed AOD values range from 0.8 to 1.0 (Note: The AOD ranges described are approximate and intended as qualitative insights from the spatial distribution plots; they do not represent computed averages or median values.). After assimilating OCM AOD, the OCMDA experiment exhibits significant improvements. The enhancements observed in OCMDA demonstrate that OCM-based assimilation effectively corrects model-simulated AOD, bringing it in line with satellite-retrieved values. The most notable improvements occur over the Central and lower Indo-Gangetic Plain (IGP), regions heavily impacted by crop residue burning in early November (Fig S1). The assimilation increases AOD by approximately 1.5 to 2.0 times compared to CNTL, highlighting the effectiveness of data assimilation in adjusting aerosol mass concentrations and refining the model’s chemical initial conditions. The larger AOD uncertainties for OCM (± 0.06 + 0.26×AOD) compared to MODIS (± 0.05 + 0.15×AOD), combined with OCM’s finer spatial resolution and stricter cloud screening, lead to more localized and noisier assimilation increments and generally cleaner retrievals, while MODIS assimilation produces smoother, often bimodal AOD patterns. To further analyze the impact of OCMDA, frequency distribution plots of AOD at 550 nm using all data from the entire Indian domain during the study period illustrate how data assimilation improves model performance (Fig. 2 (b)). The OCMDA experiment captures higher AOD frequency distributions beyond 0.5, accurately representing more intense aerosol events that CNTL often misses. In contrast, CNTL overestimates aerosol concentrations in the 0–0.5 AOD range, leading to a higher frequency distribution compared to both observed and assimilated data. This discrepancy highlights the inability of the CNTL experiment to adequately capture high aerosol loading events, whereas OCMDA demonstrates enhanced sensitivity to aerosol variability. The one-to-one comparison of OCMDA with MODISDA is presented here, while MODISDA itself has been evaluated previously in AIRWISE^13,21^, shows that improvements by OCMDA is comparable with that of MODISDA (figures from Fig. 1 (f) and Fig. 1 (i)). For instance, enhancement over IGP by OCMDA falls in close range with that by MODISDA (0.8–1.0). The bimodal distribution observed in MODISDA mainly arises from the two daily satellite overpasses (Terra in the morning and Aqua in the early afternoon), rather than from distinct aerosol types. Differences in spatial patterns between OCMDA and MODISDA can be attributed to observation timing, spatial resolution, and retrieval algorithm characteristics. In addition, OCM retrievals carry larger observation errors compared to MODIS, which leads the DA system to assign them less weight, resulting in smaller analysis increments even for similar innovations. In addition to the 10 November case, we have analysed three other high-pollution days—05, 09, and 13 November 2023—coinciding with the peak biomass burning period (Figures S14–S16, Supplementary Material). These episodes, which include some of the season’s highest observed AOD and PM₂.₅ levels, further demonstrate that OCM and MODIS AOD assimilation substantially improves the model’s ability to capture both the magnitude and spatial distribution of aerosol loading over the Indo-Gangetic Plain and Delhi.

**Fig. 3:**
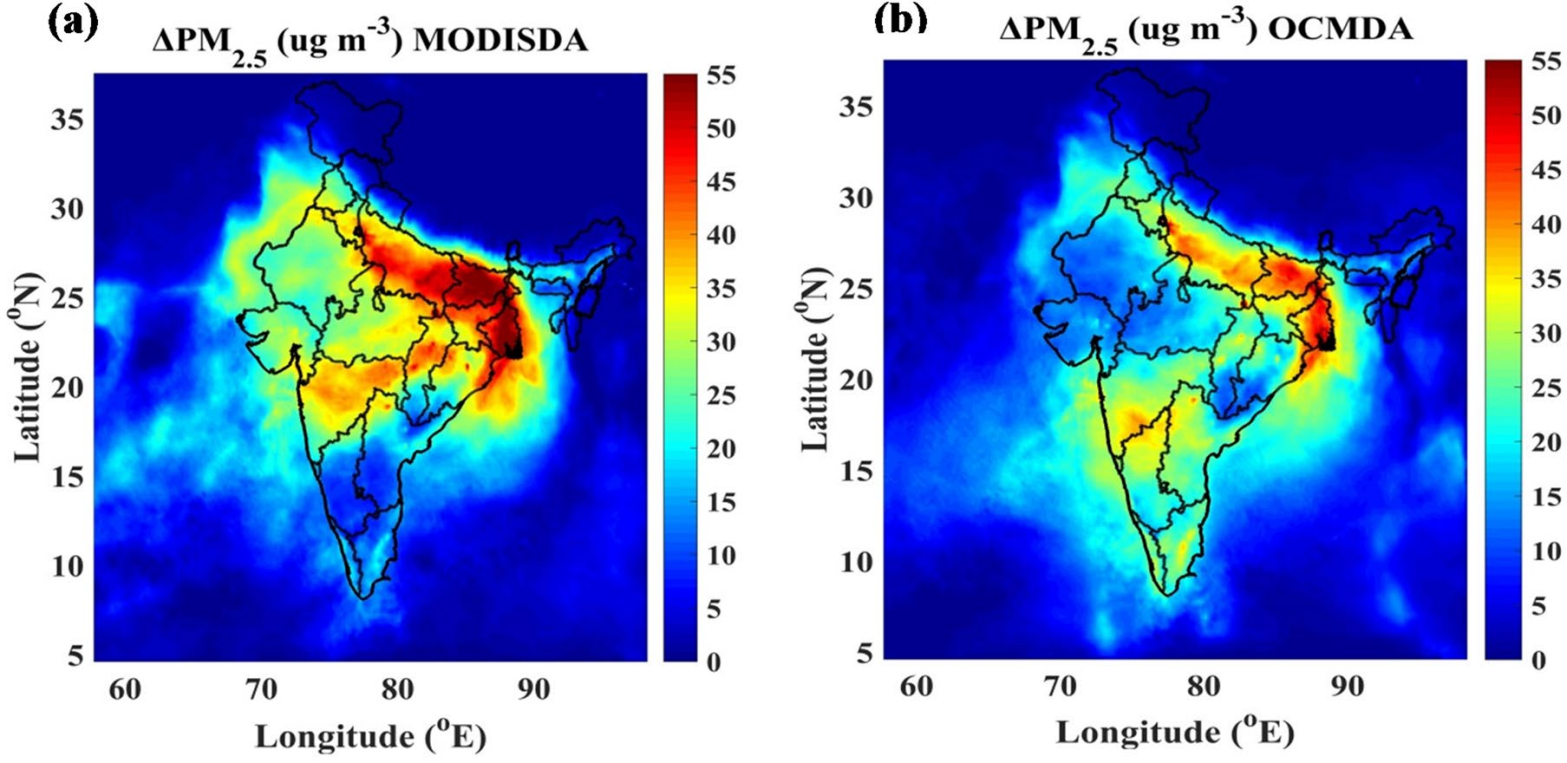
Spatial distribution of PM₂.₅ analysis increments (µg m⁻³) on 1–14 November 2023 resulting from satellite AOD assimilation. (**a**) MODIS AOD assimilation (MODISDA). and (**b**) OCM AOD assimilation (OCMDA).Analysis increments represent the difference between the assimilated and control (CNTL) simulations.

Additionally, we analyzed the impact of OCMDA on surface PM_2.5_ concentrations by comparing PM_2.5_ differences between the OCMDA and CNTL experiments, averaged over 1–14 November 2023 at the initialization of each forecast cycle (Fig. 3). On average, PM_2.5_ concentrations increased by approximately 35–45 µg m^− 3^ over the IGP due to OCM data assimilation. These increments from OCMDA are generally smaller than those from MODISDA (Fig. 3a) over the IGP region, which is likely due to the higher observational error associated with OCM. Both are generated within the GSI assimilation framework, which adjusts the mass concentrations of all aerosol species in GOCART (Goddard Global Ozone Chemistry Aerosol Radiation and Transport^22–24^ chemistry model based on assimilation-driven changes in WRF-Chem AOD. This process enhances the initial conditions for more accurate PM_2.5_ predictions.

**Fig. 4:**
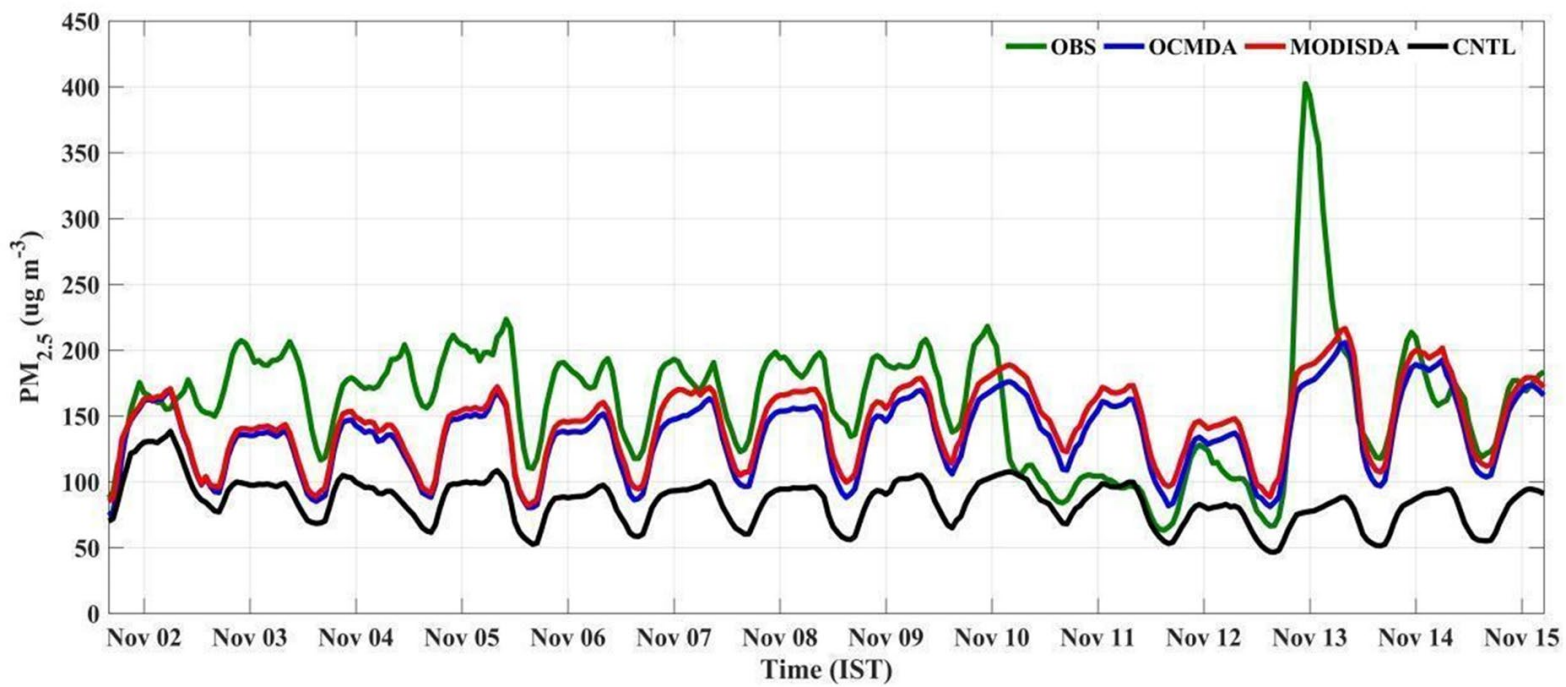
Hourly time series of surface PM₂.₅ concentrations for the first-day forecast, averaged over 258 observation sites in 12 Indian states during 1–15 November 2023.Observed concentrations (OBS) are compared with WRF-Chem simulations without assimilation (CNTL) and with assimilation of MODIS AOD (MODISDA) and OCM AOD (OCMDA).

The forecasted 72 h time series of PM_2.5_ concentrations were extracted from the CNTL, MODISDA, and OCMDA runs at 258 observation sites across 12 states of India. The average hourly PM_2.5_ values were then estimated from each of these runs and compared with the hourly mean PM_2.5_ observations across the same sites for the first day of the forecast (Fig. 4). The CNTL simulation consistently underestimates PM_2.5_ concentrations throughout the period, while both the OCMDA and MODISDA significantly improves agreement with observations. The improvements in OCMDA and MODISDA highlight the effectiveness of satellite AOD assimilation in enhancing model performance, particularly during the biomass burning period during the first ten days of November. A sharp decrease in PM_2.5_ concentration was observed between 10 and 11 November, attributed to rainfall in parts of India. However, limitations in satellite AOD retrieval during cloud cover affected the dataset. Despite this, both assimilation runs successfully captured the overall trend of decreasing aerosol concentrations, as reflected in the observed data on 12 November. MODISDA shows slightly higher PM_2.5_ concentrations compared to OCMDA with the average difference between the two assimilation runs being relatively small, around 8 µg m^− 3^. The impact of assimilation is also analysed for localized events during the simulation period. For example, a sharp spike in PM_2.5_ concentrations was observed in the early morning of 13 November, following the Diwali celebrations on 12 November, where the observed average PM_2.5_ levels reached over 400 µg m^− 3^. This increase may be attributed to the use of firecrackers during the Diwali festival across India, even after the ban on firecracker use is imposed in major Indian cities. The influence of firecracker usage is evident in the elevated PM_2.5_ pollution observed in multiple regions. OCMDA simulations show the tendency to capture this event, although with an underestimation of approximately 200 µg m^− 3^, whereas the CNTL simulation completely missed this episode. Similar tendency is noticed in MODISDA time series for this event. This underperformance highlights the localized nature of the event, which presents challenges for capturing such spikes in regional-scale models. Nevertheless, the assimilation of MODIS and OCM AOD data significantly improves PM_2.5_ predictions compared to the CNTL run. Also the regional analysis (Supplementary Figures S3–S13) shows that the impact of DA varies across states. Urban and high-emission regions such as Delhi, West Bengal, and Punjab display marked improvements in peak pollution capture and bias reduction, while rural or lower-emission states like Odisha and parts of Madhya Pradesh also show positive, though relatively moderate, improvements. These results highlight spatial heterogeneity in DA performance, influenced by differences in local emissions, meteorology, and observational coverage.

The performance of all three experimental setups, OCMDA, MODISDA, and CNTL, is systematically assessed using key statistical metrics: RMSE (µg m⁻³), MB (µg m⁻³), and R (Table 1). This evaluation aims to quantify the impact of OCM-based data assimilation on the accuracy and reliability of air quality forecasts across multiple regions. Additionally, we have compared the OCMDA experiment with MODISDA to assess whether OCMDA is comparable in performance. Overall, the application of OCM data assimilation significantly improved the forecasts compared to the CNTL simulation, demonstrating notable enhancements in RMSE, MB, and R across the states. These improvements vary with geographic and pollution regimes: larger gains are generally seen in high-pollution Indo-Gangetic Plain (IGP) states, where strong aerosol loading provides a clearer assimilation signal, whereas improvements in cleaner or more meteorologically complex coastal and central states tend to be more modest and sometimes decrease more quickly with forecast lead time. These improvements highlight the effectiveness of assimilating OCM AOD in refining model predictions and reducing biases in simulated PM_2.5_ concentrations.

To provide context for the MB values in Table 1, we also report the mean observed PM₂.₅ for each state and forecast day (Supplementary Table S4). This addition highlights, for example, that a 40 µg m⁻³ bias in Delhi corresponds to an observed mean of ~ 250 µg m⁻³, while in Gujarat the same bias represents a much larger fraction of the observed mean (~ 95 µg m⁻³). Similarly, in Bihar and Uttar Pradesh, where mean observed concentrations exceed 125 µg m⁻³, reductions in MB of 15–20 µg m⁻³ translate into meaningful improvements in forecast skill but still leave substantial residual pollution levels. By contrast, in relatively cleaner states such as Maharashtra (mean ~ 70–75 µg m⁻³) and Rajasthan (~ 98 µg m⁻³), even small absolute biases represent a large relative error, underscoring the difficulty of accurately simulating lower-concentration environments. These comparisons show that the impact of DA must be judged relative to the prevailing air quality regime: in high-pollution Indo-Gangetic Plain states, DA improves the capture of extreme events, while in lower-pollution or more variable meteorological regimes, improvements are more subtle but equally important for meeting air-quality standards.

**Table 1:** Statistical evaluation of PM_2.5_ forecasts for day 1, day 2, and day 3 across 12 Indian States.

Forecast	CNTL			MODISDA			OCMDA		
	RMSE	MB	R	RMSE	MB	R	RMSE	MB	R
**Bihar**									
Day 1	62.44	-43.4	0.02	42.42	17.84	0.49	39.35	6.48	0.45
Day 2	64.47	-45.8	-0.1	42.86	13.24	0.36	41.29	4.46	0.33
Day 3	66.11	-49.1	-0	40.62	7.194	0.33	40.22	1.28	0.31
**Delhi**									
Day 1	129.55	-69.0	0.4	127.27	49.78	0.48	121.78	40.51	0.50
Day 2	131.65	-74.5	0.44	136.74	44.96	0.47	133.08	38.47	0.48
Day 3	125.63	-74.9	0.52	122.77	39.90	0.57	117.65	31.84	0.59
**Gujarat**									
Day 1	35.18	-24.3	0.51	29.84	21.67	0.72	21.35	9.09	0.75
Day 2	36.09	-25.3	0.49	30.21	21.76	0.71	21.47	8.63	0.74
Day 3	37.13	-25.7	0.47	31.09	22.11	0.7	22.75	9.83	0.73
**Haryana**									
Day 1	100.65	-83.7	0.49	63.34	-31.22	0.50	66.10	-36.72	0.51
Day 2	105.61	-89.4	0.57	71.83	-40.57	0.44	74.03	-45.5	0.46
Day 3	108.19	-94.2	0.71	73.45	-49.55	0.58	75.64	-53.56	0.60
**Jharkhand**									
Day 1	47.37	-15.4	0.38	68.34	51.72	0.46	60.73	2.67	0.47
Day 2	50.85	-21.6	0.32	59.87	39.23	0.38	55.36	-3.31	0.54
Day 3	51.76	-22.0	0.27	59.66	33.69	0.22	57.82	-7.72	0.54
**Maharashtra (Pune)**									
Day 1	66.32	-40	0.3	49.18	4.81	0.49	48.70	2.67	0.47
Day 2	68.64	-42.6	0.37	50.01	-1.46	0.49	48.46	-3.31	0.54
Day 3	71.75	-45.2	0.38	50.20	-5.18	0.54	50.76	-7.72	0.54
**Madhya Pradesh**									
Day 1	52.21	-27.9	0.19	41.33	16.53	0.54	38.04	4.14	0.55
Day 2	54.16	-28.6	0.15	44.10	14.46	0.43	41.00	4.63	0.47
Day 3	56.44	-30.6	0.14	44.12	10.69	0.43	42.55	3.20	0.46
**Odisha**									
Day 1	63.49	-44.6	0.6	34.02	8.65	0.80	38.04	4.14	0.55
Day 2	65.61	-48.2	0.7	36.24	9.80	0.78	41.00	4.63	0.47
Day 3	72.22	-54.6	0.5	40.10	1.48	0.67	42.55	3.20	0.46
**Punjab**									
Day 1	85.41	-65	0.3	59.72	-31.33	0.45	62.01	-35.1	0.44
Day 2	87.17	-67.0	0.3	63.47	-34.81	0.43	64.39	-37.0	0.44
Day 3	86.71	-65.8	0.3	62.65	-33.45	0.49	63.71	-35.6	0.49
**Rajasthan**									
Day 1	64.8	-45.1	-0.01	42.11	-9.88	0.34	46.63	-21.2	0.31
Day 2	66.52	-48.5	0.07	44.99	-13.8	0.30	48.59	-23.4	0.32
Day 3	70.55	-54.0	0.23	48.65	-23.12	0.40	52.27	-30.4	0.42
**Uttar Pradesh**									
Day 1	66.00	-44.0	0.3	47.83	18.53	0.53	46.20	10.62	0.505
Day 2	66.24	-43.1	0.3	53.78	19.60	0.41	51.23	13.86	0.43
Day 3	67.45	-46.6	0.4	48.80	11.41	0.51	47.08	7.096	0.536
**West Bengal (Kolkata)**									
Day 1	87.22	-71.5	0.5	64.08	28.91	0.64	60.76	18.47	0.60
Day 2	75.7	-55.4	0.6	75.60	49.24	0.70	73.18	41.74	0.65
Day 3	82.57	-62.0	0.5	75.21	40.41	0.63	74.14	34.18	0.59

In Bihar, OCMDA significantly reduced both the mean bias (MB) and root mean square error (RMSE) compared to the control (CNTL) simulation, achieving improvements comparable to those observed with MODISDA. For instance, on Day 1, the RMSE in the CNTL run was substantially high, but OCMDA reduced it by nearly 20 µg m⁻³, which is similar to the reduction achieved by MODISDA. Furthermore, while OCMDA demonstrated the lowest MB and RMSE among all configurations and its correlation coefficient (R) was comparable to that of MODISDA across most regions and forecast days; differences in R were generally small.

In Delhi, the CNTL simulation consistently underestimated PM_2.5_ concentrations, particularly during periods of high pollution. Both OCMDA and MODISDA addressed this underestimation, with OCMDA showing a slightly better correlation (*R* = 0.5) on Day 1, indicating its effectiveness in aligning forecasts with observations.

For Gujarat, OCMDA demonstrated substantial improvements over CNTL, reducing RMSE values for Day 1 from 35.18 µg m⁻³ in CNTL to 21.35 µg m⁻³, outperforming MODISDA, which achieved 29.84 µg m⁻³. Similarly, correlation (R) values improved from 0.51 in CNTL to 0.75 in OCMDA and 0.72 in MODISDA, reflecting a stronger agreement with observed PM_2.5_ concentrations.

A similar trend was observed in Haryana, where both assimilation experiments significantly improved RMSE and MB compared to CNTL, although a slight underestimation persisted in both MODISDA and OCMDA.

For states like Maharashtra (Pune) and Madhya Pradesh, MODISDA slightly outperformed OCMDA in reducing RMSE on Day 1, although the difference between the two was minimal. In Punjab and Rajasthan, MODISDA exhibited better performance, particularly in capturing PM_2.5_ variability during pollution episodes such as Diwali. However, OCMDA also showed significant improvements, effectively reducing errors and improving correlation coefficients.

In Rajasthan, Uttar Pradesh, and Punjab both assimilation experiments demonstrated the clear benefits of data assimilation in enhancing air quality forecasts. OCMDA and MODISDA consistently reduced RMSE and improved correlation (R) compared to CNTL, indicating a better alignment between forecasts and observed trends. In Rajasthan, MODISDA significantly outperformed CNTL by correcting its poor correlation and large RMSE values. Similarly, in Uttar Pradesh, both OCMDA and MODISDA exhibited substantial improvements, with OCMDA slightly surpassing MODISDA in reducing RMSE for certain days. Punjab followed a similar pattern, where both assimilation experiments corrected CNTL’s large biases and weak correlations, particularly in the early forecast periods. In Jharkhand, MODISDA significantly degraded the performance in terms of both MB and RMSE, despite showing relatively strong short-term forecast skill. Similarly, OCMDA also led to a slight degradation in RMSE; however, it notably improved the MB, indicating better alignment with observed concentration levels. Moreover, OCMDA achieved a higher correlation coefficient (*R* = 0.5446) over extended forecast periods, highlighting its potential to enhance the temporal evolution of PM₂.₅ predictions through improved assimilation of OCM aerosol observations. A closer look at the MB values in Table 1 reveals distinct regional patterns. OCMDA tends to produce lower MB than MODISDA in Jharkhand, Bihar, and Madhya Pradesh, while both DA runs show similar MB in Uttar Pradesh, Maharashtra, and Odisha. In Punjab and Rajasthan, OCMDA’s MB is slightly higher than that of MODISDA. These differences may reflect regional variations in AOD retrieval accuracy due to land surface type, with bright or heterogeneous surfaces in semi-arid and agricultural regions increasing retrieval uncertainty. They may also be influenced by the timing and persistence of cloud cover, which can limit AOD data availability for assimilation, and by differences in aerosol chemical composition, which affect the relationship between column AOD and surface PM₂.₅.

Across all states, the Day 1 forecast consistently showed better performance compared to the second day (Day 2) and third day (Day 3) forecasts, as RMSE values increased and correlation coefficients slightly decreased with increasing lead time. For example, in Odisha, OCMDA achieved an RMSE of 34.02 µg m⁻³ and *R* = 0.80 for Day 1, while RMSE increased to 36.32 µg m⁻³ and *R* = 0.78 by Day 2. Similarly, in West Bengal (Kolkata), OCMDA reduced Day 1 RMSE to 64.08 µg m⁻³ compared to CNTL’s 87.22 µg m⁻³, with R improving to 0.64 from 0.56. These examples underline that while both assimilation methods improve forecasts across all regions, the scale of improvement is influenced by geographic setting (urban vs. rural), pollution regime (high-pollution IGP vs. cleaner/coastal states), and the strength of the AOD signal. To support these statistical evaluations, time series of PM₂.₅ concentrations for each state have been plotted and are provided in the supplementary figures (S3 to S13). We also provide a supplementary AQI skill-score table (Supplementary Table S1) that reports category-wise Accuracy, False Alarm Ratio (FAR), Probability of Detection (POD) and Critical Success Index (CSI) for CNTL, MODISDA and OCMDA (Day-1 to Day-3). Supplementary Table S1 highlights regional contrasts — for example, large POD/CSI gains from DA in the IGP (Bihar, Delhi, Punjab) and cases where OCMDA better captures localized urban peaks while MODISDA shows stronger performance for broad regional accumulation. Entries where no event was present are marked NAN.

Overall, the comparison between OCMDA and MODISDA confirms that OCMDA is highly effective in improving PM_2.5_ forecasts and, in some cases, performs comparably to or better than MODISDA, further validating the value of OCM-based data assimilation in air quality modeling.

Thus, the improvements in statistical parameters decrease with increasing forecast lead time. This indicates that model deficiencies, such as uncertainties in emissions, meteorology, or chemical processes, begin to offset the benefits of AOD assimilation over time. Independent observations of the vertical aerosol distribution and chemical composition were not available for the study period. Although our operational archive for this experiment did not retain the 3-D aerosol fields required to reconstruct vertical AOD profiles, future analyses should exploit model-based profiles (e.g., fraction of column AOD within the lowest 1 km) to distinguish surface-dominant from elevated layers and to interpret regional differences in DA performance.The uncertainty of the OCM sensor, as calculated by the SAER algorithm, is higher than that of MODIS, indicating relatively larger retrieval errors in OCM-derived AOD. Additionally, OCM as of now does not provide data over the ocean region. If such data were available, it would allow for the assimilation of dust plumes originating from the Middle East and transported over the sea, thereby improving the representation of their impact on regional air quality. Furthermore, since MODIS fire count data will not be available in the future, having fire count data from the Oceansat satellite would be a valuable asset for air quality forecasting, ensuring continuity in fire emissions monitoring and improving model accuracy. Additionally, while the spatial resolution of OCM data is generally sufficient for broad-scale applications, its finer resolution enhances detailed, city-centric forecasting. However, its effectiveness may be influenced by temporal frequency and retrieval accuracy, particularly under challenging atmospheric conditions such as high aerosol loading or cloud cover. To address these challenges and fully exploit the advantages of OCM data for urban areas, future research should explore the benefits of integrating OCM data into higher-resolution model configurations. This advancement could significantly refine our simulation capabilities, enabling more precise predictions of aerosol dynamics and interactions. By focusing on urban environments, where aerosol impacts are often most critical, the assimilation of OCM data can lead to improved forecast accuracy that is vital for effective air quality management in metropolitan areas.

## **Conclusion**

The OCM refers to the Ocean Colour Monitor sensor aboard India’s Oceansat-3 satellite, providing AOD at a high-resolution of 770 m since January 2023. The present study, for the first time, evaluated the utilization of AOD retrievals of OCM in the chemical data assimilation framework of AIRWISE for improving PM₂.₅ air quality forecasts across India. The study is conducted during 1st -15th November 2023, keeping the entire India and surrounding region as the study domain. All simulation experiments provide PM_2.5_ forecast three days in advance. For evaluating the impact of OCM AOD assimilation, three sets of simulation experiments are conducted (i) Without AOD assimilation (CNTL), (2) With OCM AOD assimilation (OCMDA) and (3) With MODIS AOD assimilation (MODISDA).The research highlights significant improvements in forecast accuracy during the selected high-pollution period. Assimilating OCM AOD data significantly enhanced WRF-Chem-simulated AOD, bringing values closer to observations (0.8–1.0) compared to the underestimated CNTL range (0.1–0.4). The improvements in OCMDA closely match those of MODISDA, demonstrating that OCM-based assimilation is as effective as MODIS in refining aerosol distributions.

The state-wise analysis of Indian states based on ground observations of PM_2.5_ revealed significant reductions in RMSE and MB in PM₂.₅ forecasts underscoring the significance of OCMDA. For example, in Bihar, the Day 1 RMSE dropped from 62.44 µg/m³ in CNTL to 39.36 µg/m³ with OCMDA, showcasing the effectiveness of OCM assimilation. Similarly, in Gujarat, the RMSE for Day 1 forecasts improved from 35.18 µg/m³ in CNTL to 21.36 µg/m³ with OCMDA. Additionally, correlation coefficients (R) increased from 0.51 in CNTL to 0.75 in OCMDA, demonstrating a stronger alignment with observed PM₂.₅ concentrations.

Due to assimilation significant reduction in underestimation over Haryana and Punjab, along with a noticeable sign change in mean bias over Delhi, highlights the role of local emission uncertainties emphasizing the importance of region-specific emission characterization for improving forecast accuracy.

The statistical analysis shows that assimilating OCM AOD is equally good as that of MODIS AOD. These findings confirm that OCM-based AOD assimilation substantially improves air quality forecasts, performing on par with or even exceeding MODIS-based assimilation in certain regions. This reinforces the potential of OCM AOD as a reliable data source for enhancing regional air quality predictions through chemical data assimilation.

Both assimilation experiments successfully captured major pollution events, such as the Diwali-induced PM₂.₅ spike on November 12, 2023, when observed concentrations exceeded 400 µg/m³. This finding demonstrates the ability of data assimilation to significantly enhance the model’s capability to capture regional trends in PM₂.₅ pollution, though localized, short-term events remain challenging.

A key observation is that the benefits of AOD assimilation diminish with increasing forecast lead times, highlighting the growing influence of model uncertainties over longer time horizons. Moreover, the MODISDA and OCMDA experiments effectively capture higher frequency distributions beyond 0.5 AOD, reflecting their enhanced capability to accurately track more intense aerosol events, which are often missed by the CNTL. To further enhance PM_2.5_ predictions, leveraging finer resolution data from the OCM and assimilating it into a higher-resolution, city-specific model configuration could be particularly beneficial. Overall, this study underscores the critical role of data assimilation in improving air quality forecasts, with the OCM sensor emerging as a promising alternative to MODIS. The results validate the potential of OCM data to ensure the continuity and advancement of air quality forecasting systems in India, enabling more accurate and actionable predictions for mitigating pollution episodes.

## Supplementary Information

Below is the link to the electronic supplementary material.

Supplementary Material 1

## Data Availability

The PM2.5 observational data used in this study were obtained from the Central Pollution Control Board (CPCB) and are accessible at https://app.cpcbccr.com/ccr. MODIS Aerosol Optical Depth (AOD) retrievals were downloaded from NASA Earthdata at https://earthdata.nasa.gov/. OCM data were acquired from the Space Applications Centre (SAC), ISRO, and are available at https://www.mosdac.gov.in/.
