# Supplementary material for: Assimilation of high-resolution Ocean Color Monitor (OCM) aerosol optical depth in WRF-Chem improves PM₂.₅ forecasts over the Indian region
Source: Sci Rep. 2025 Nov 12;15:39669. doi: 10.1038/s41598-025-23307-1 (PMC12612248; doi:10.1038/s41598-025-23307-1)
Supplement: Supplementary file 1 — Supplementary Material 1 [file 41598_2025_23307_MOESM1_ESM.docx]

**Supplementary material**

**Assimilation of high-resolution Ocean Color Monitor (OCM) aerosol optical depth in WRF-Chem improves PM₂.₅ forecasts over the Indian region.**

Prafull P. Yadav^1, 2*^, Sachin D.Ghude^1*^, Rajesh Kumar^4^, Gaurav Govardhan^1, 3^, Rajmal Jat^1^, Shivani Shah^5^, B. P. Shukla^5^, Manoj K. Mishra^5^, Deepak Putrevu^5^, , P. K. Thapliyal^5^, Rashmi Sharma^5^

^1^Indian Institute of Tropical Meteorology, Ministry of Earth Sciences, Pune, India

^2^Dept. of Atmospheric and Space Sciences, Savitribai Phule Pune University, Pune, India

^3^National Center for Medium Range Weather Forecasting, Ministry of Earth Sciences, Noida, UP, India

^4^National Science Foundation National Center for Atmospheric Research, Boulder, Colorado, USA

^5^Space Applications Centre, Ahmedabad, India

*Corresponding Authors: Sachin D. Ghude, Prafull P. Yadav

E-mail address: [sachinghude@tropmet.res.in](mailto:sachinghude@tropmet.res.in), [prafull.yadav@tropmet.res.in](mailto:prafull.yadav@tropmet.res.in)


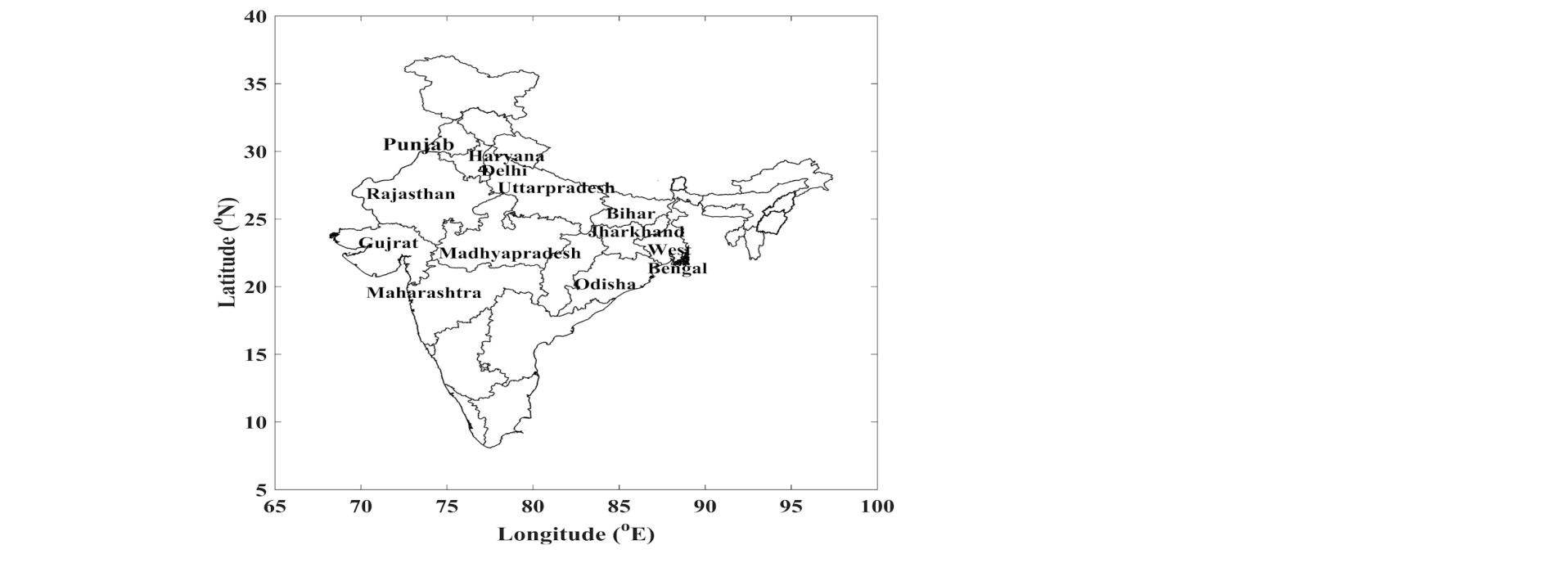


Figure S1. Geographic locations of the twelve selected Indian states considered in this study.


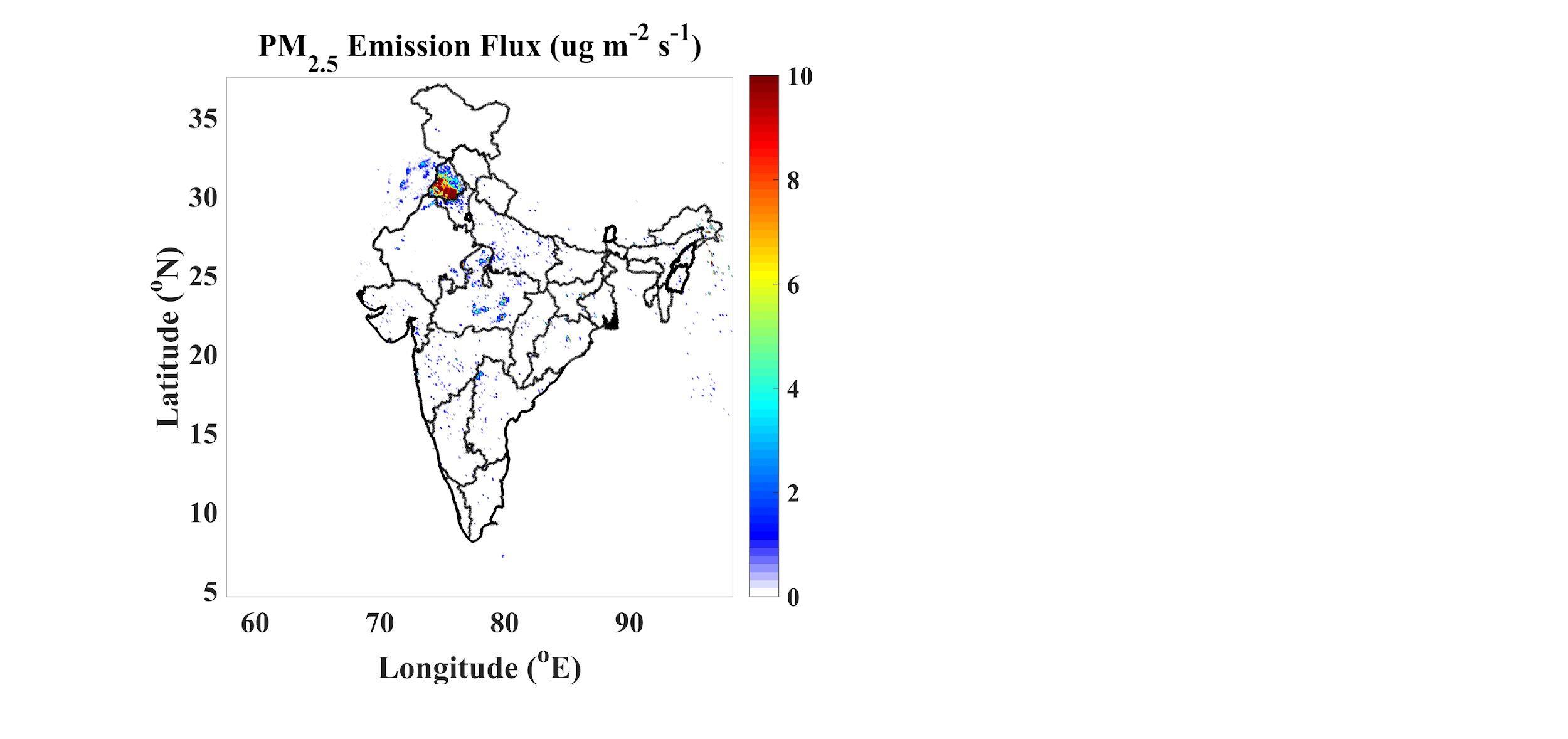


Figure S2: PM_2.5_ emission flux from fires (averaged over 13 days).


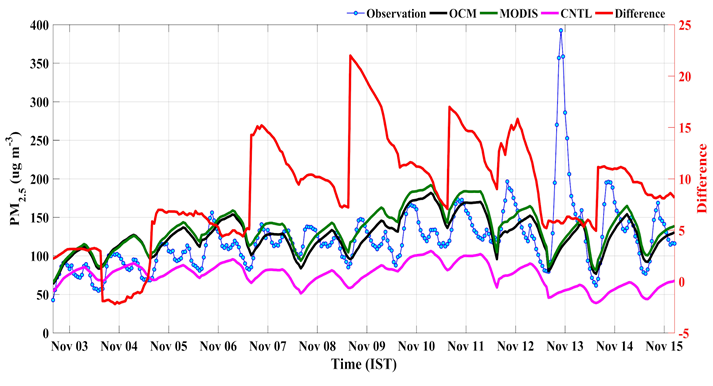


**(b)**


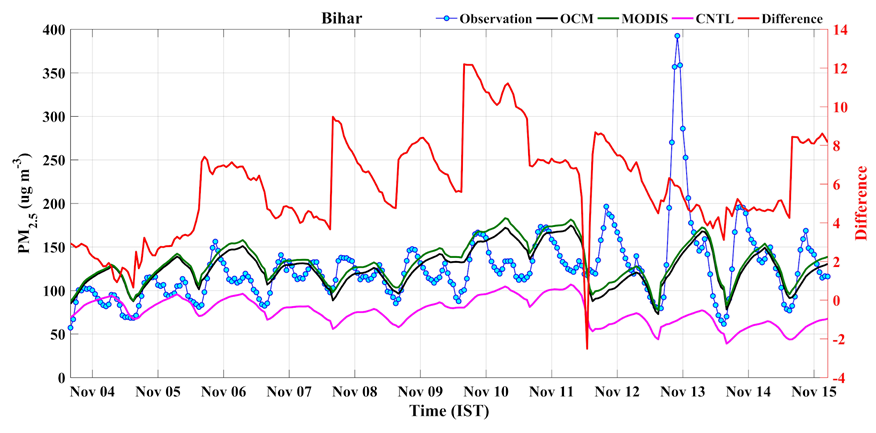


**(c)**


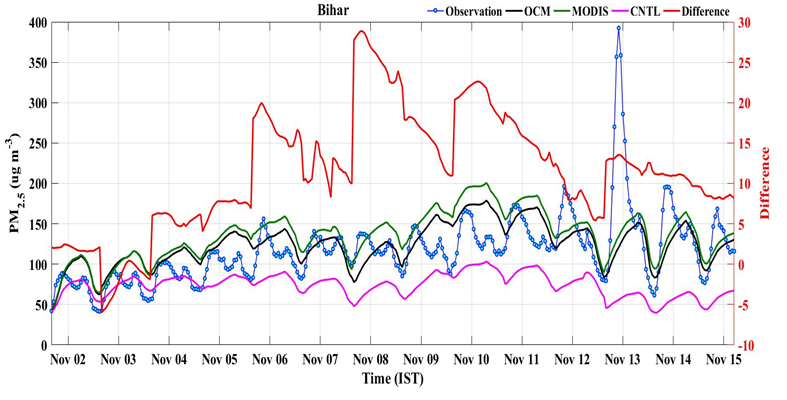


**(a)**

Figure S3. Time series of observed and WRF-Chem forecasted hourly PM₂.₅ mass concentrations in Bihar for the (a) first, (b) second, and (c) third forecast days. The left Y-axis represents PM₂.₅ concentrations (µg m⁻³), while the right Y-axis indicates the absolute difference between MODISDA and OCMDA forecasts. The line plots show observations (blue), CNTL (pink), MODIS (MODISDA, green), and OCM (OCMDA, black). The red line represents the MODISDA–OCMDA difference.


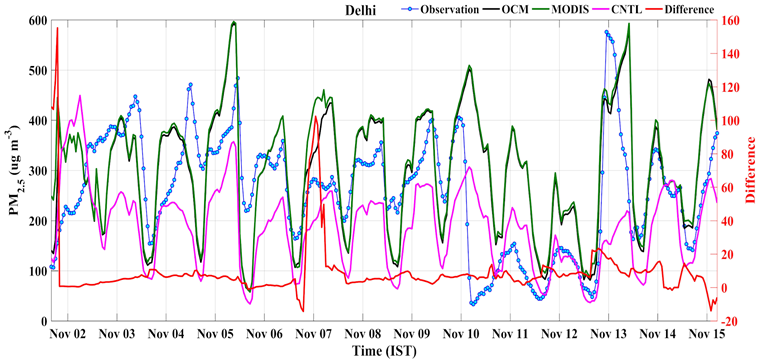


**(a)**


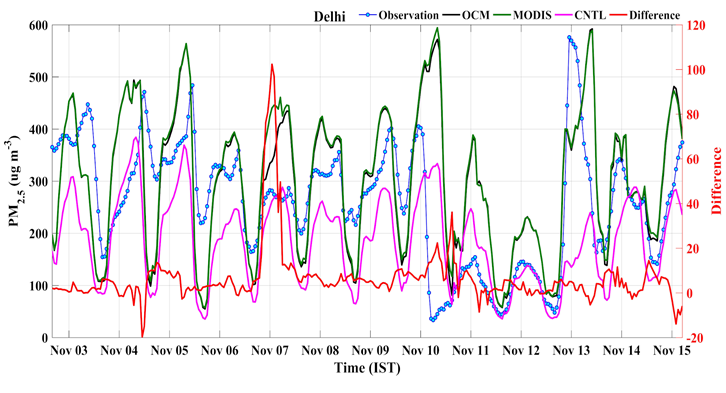


**(b)**


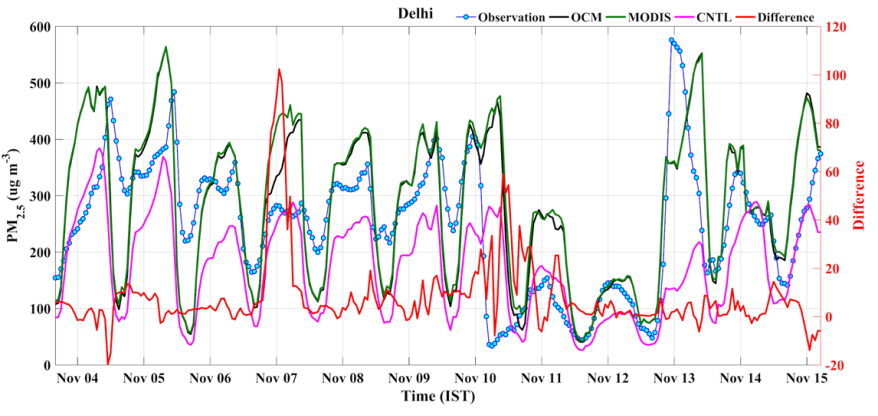


**(c)**

Figure S4. Time series of observed and WRF-Chem forecasted hourly PM₂.₅ mass concentrations in Delhi for the (a) first, (b) second, and (c) third forecast days. The left Y-axis represents PM₂.₅ concentrations (µg m⁻³), while the right Y-axis indicates the absolute difference between MODISDA and OCMDA forecasts. The line plots show observations (blue), CNTL (pink), MODIS (MODISDA, green), and OCM (OCMDA, black). The red line represents the MODISDA–OCMDA difference.


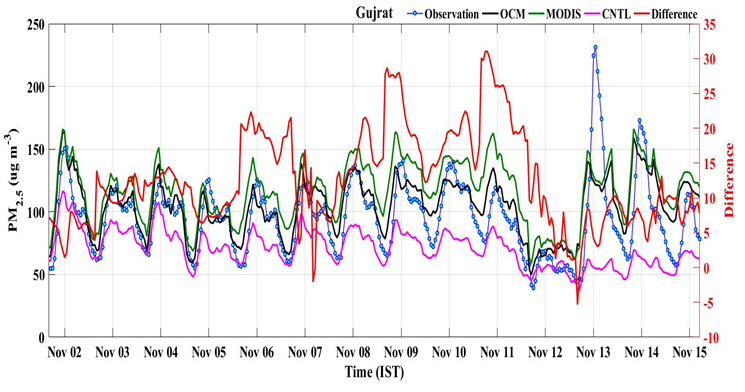


**(a)**


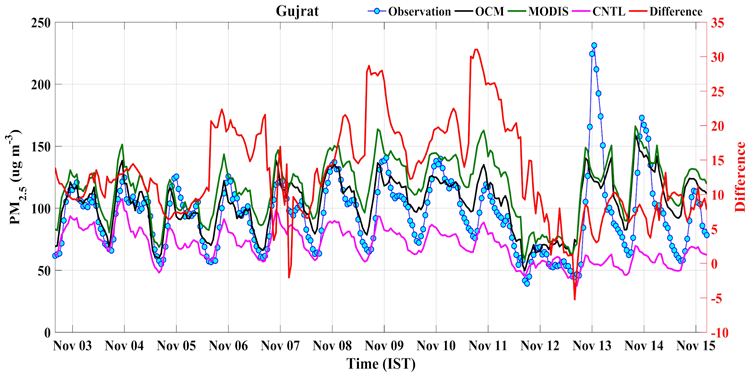


**(b)**


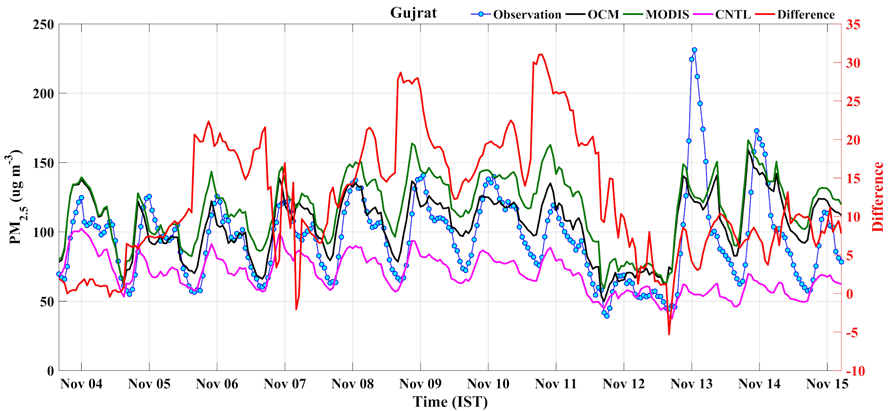


**(c)**

Figure S5. Time series of observed and WRF-Chem forecasted hourly PM₂.₅ mass concentrations in Gujrat for the (a) first, (b) second, and (c) third forecast days. The left Y-axis represents PM₂.₅ concentrations (µg m⁻³), while the right Y-axis indicates the absolute difference between MODISDA and OCMDA forecasts. The line plots show observations (blue), CNTL (pink), MODIS (MODISDA, green), and OCM (OCMDA, black). The red line represents the MODISDA–OCMDA difference.

**(b)**


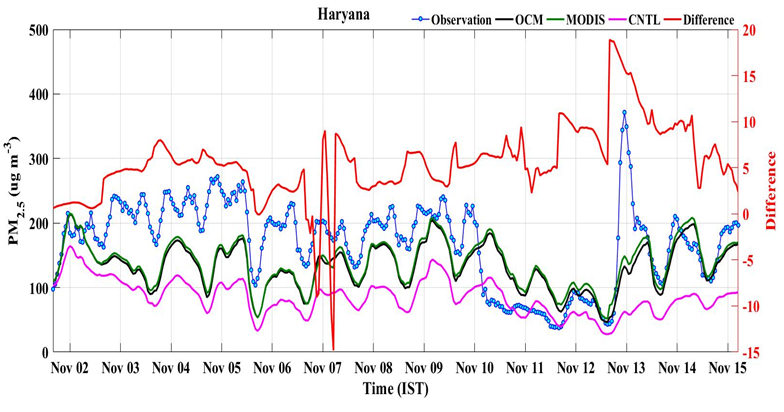


**(a)**


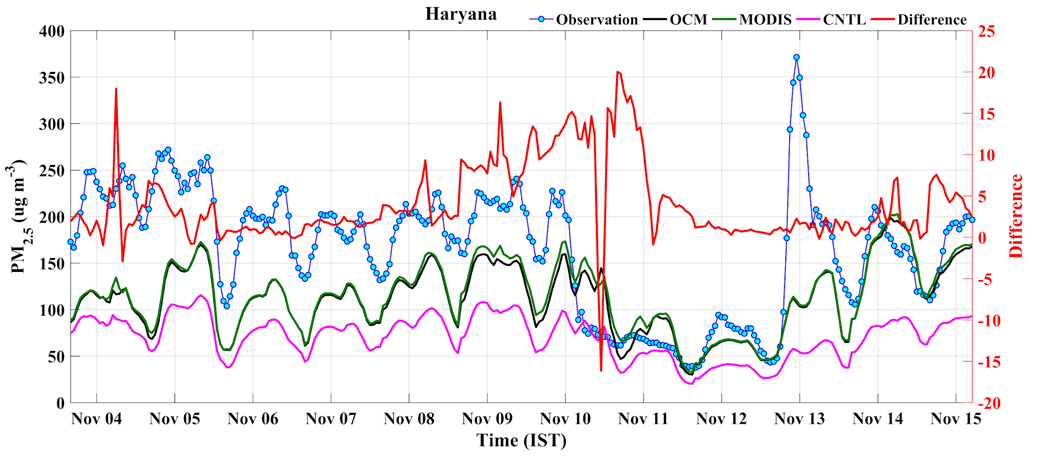


**(c)**


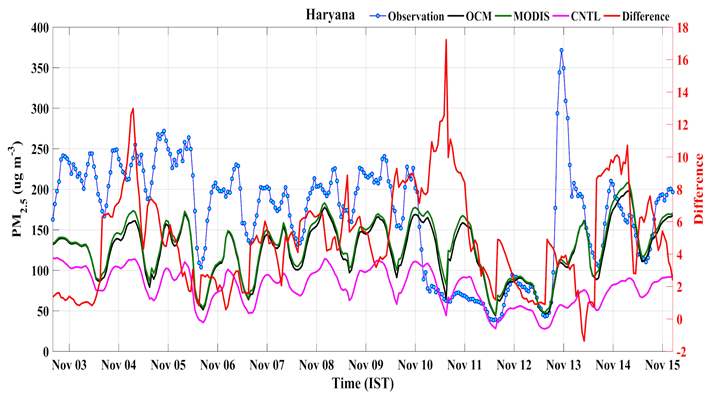


**(b)**

Figure S6. Time series of observed and WRF-Chem forecasted hourly PM₂.₅ mass concentrations in Haryana for the (a) first, (b) second, and (c) third forecast days. The left Y-axis represents PM₂.₅ concentrations (µg m⁻³), while the right Y-axis indicates the absolute difference between MODISDA and OCMDA forecasts. The line plots show observations (blue), CNTL (pink), MODIS (MODISDA, green), and OCM (OCMDA, black). The red line represents the MODISDA–OCMDA difference.

**(b)**


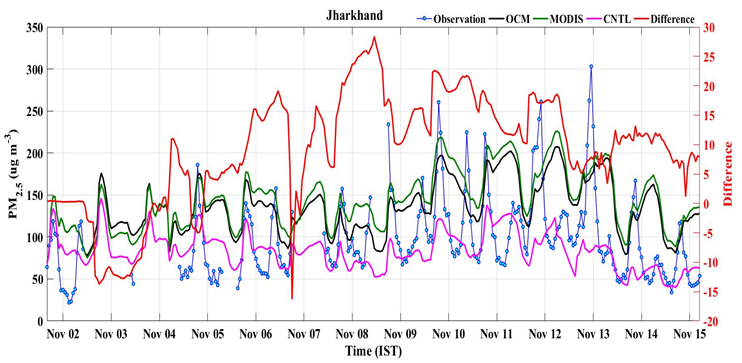


**(a)**


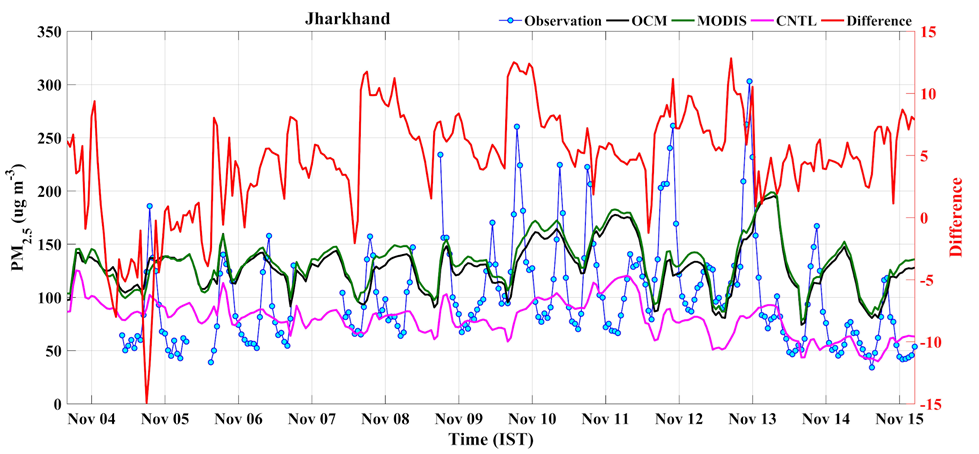


**(c)**


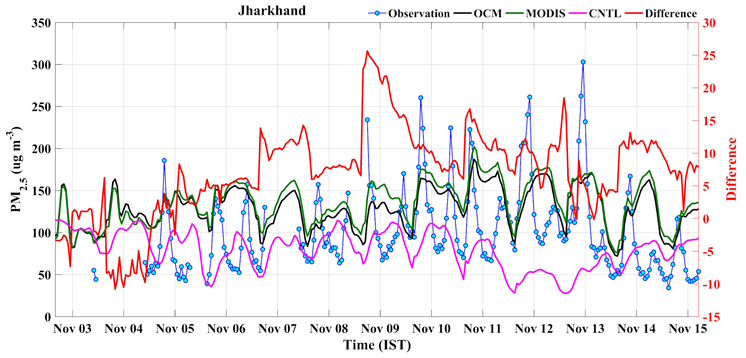


**(b)**

Figure S7. Time series of observed and WRF-Chem forecasted hourly PM₂.₅ mass concentrations in Jharkhand for the (a) first, (b) second, and (c) third forecast days. The left Y-axis represents PM₂.₅ concentrations (µg m⁻³), while the right Y-axis indicates the absolute difference between MODISDA and OCMDA forecasts. The line plots show observations (blue), CNTL (pink), MODIS (MODISDA, green), and OCM (OCMDA, black). The red line represents the MODISDA–OCMDA difference.

**(a)**


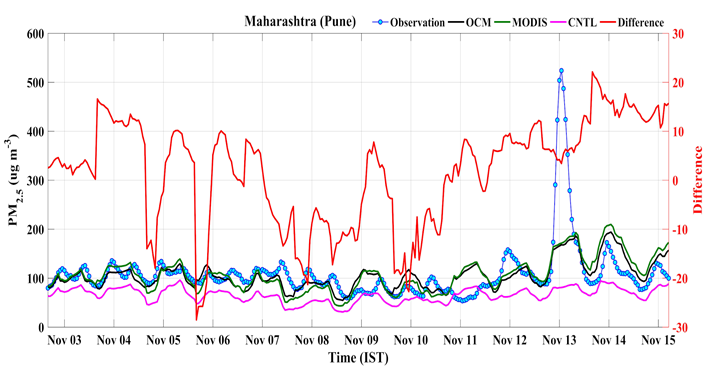


**(b)**


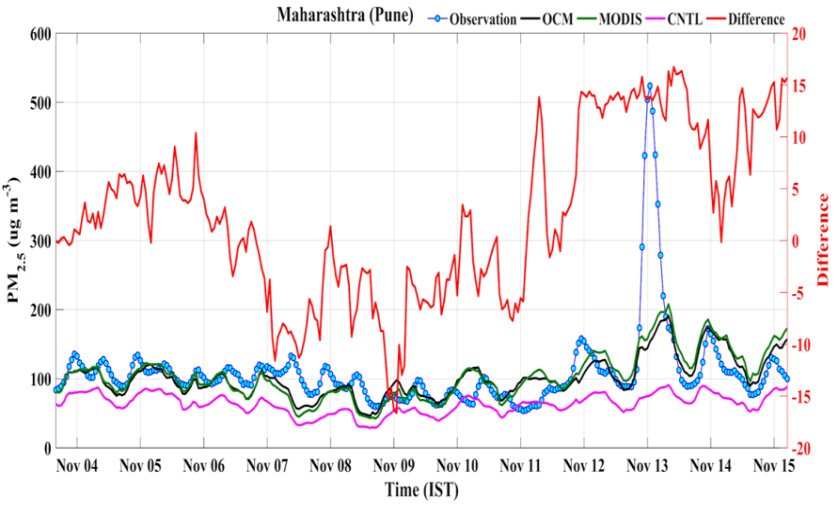


**(c)**


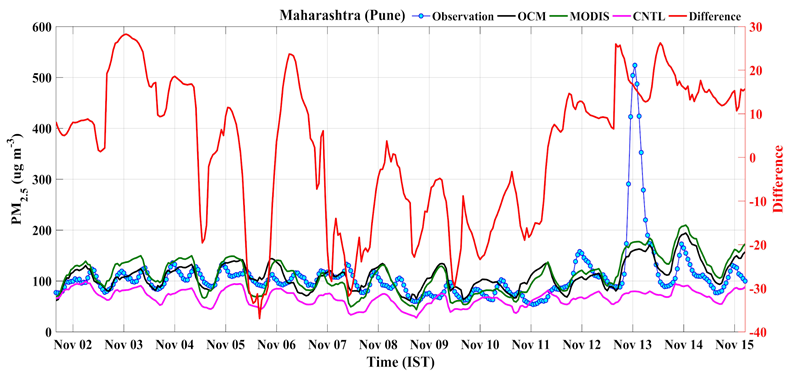


**(a)**

Figure S8. Time series of observed and WRF-Chem forecasted hourly PM₂.₅ mass concentrations in Maharashtra (Pune) for the (a) first, (b) second, and (c) third forecast days. The left Y-axis represents PM₂.₅ concentrations (µg m⁻³), while the right Y-axis indicates the absolute difference between MODISDA and OCMDA forecasts. The line plots show observations (blue), CNTL (pink), MODIS (MODISDA, green), and OCM (OCMDA, black). The red line represents the MODISDA–OCMDA difference.

**(a)**


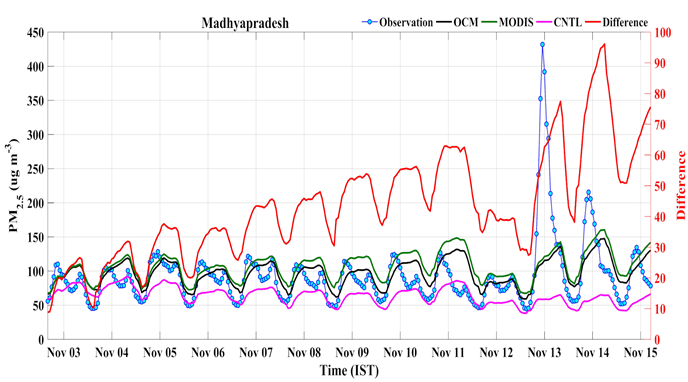


**(b)**


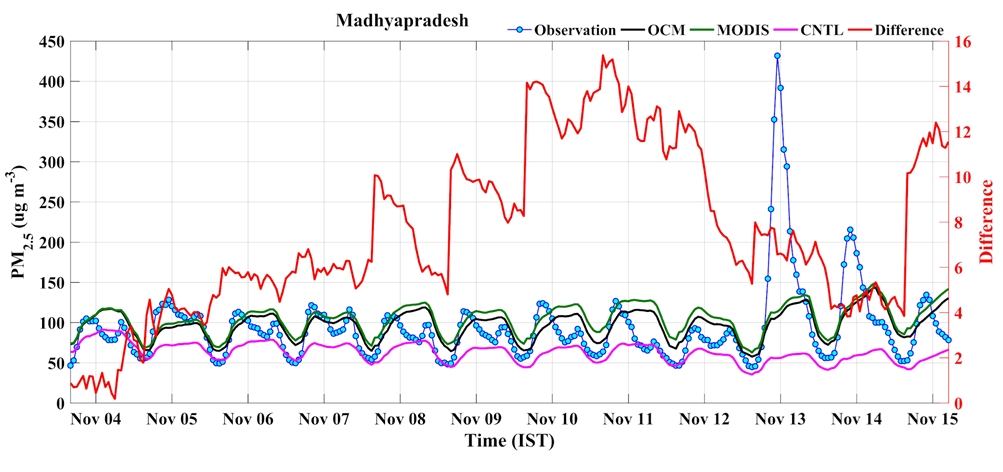


**(c)**


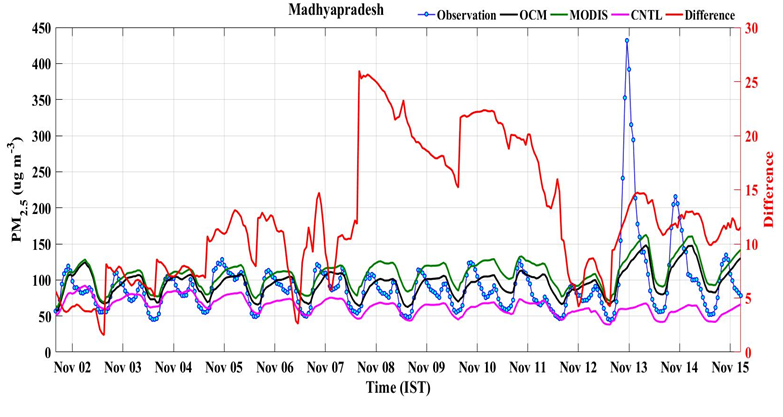


**(a)**

Figure S9. Time series of observed and WRF-Chem forecasted hourly PM₂.₅ mass concentrations in Madhyapradesh for the (a) first, (b) second, and (c) third forecast days. The left Y-axis represents PM₂.₅ concentrations (µg m⁻³), while the right Y-axis indicates the absolute difference between MODISDA and OCMDA forecasts. The line plots show observations (blue), CNTL (pink), MODIS (MODISDA, green), and OCM (OCMDA, black). The red line represents the MODISDA–OCMDA difference.

**(b)**


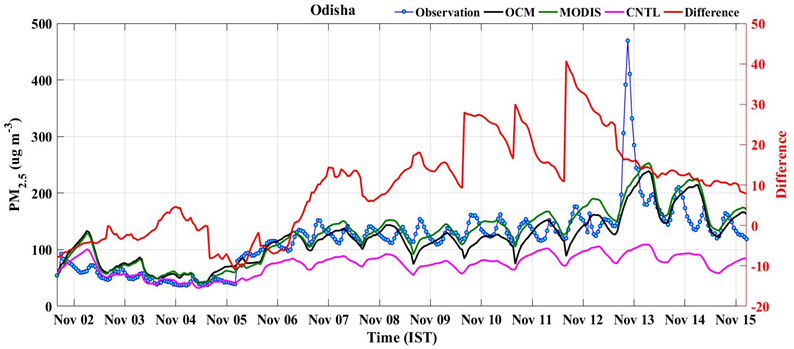


**(a)**


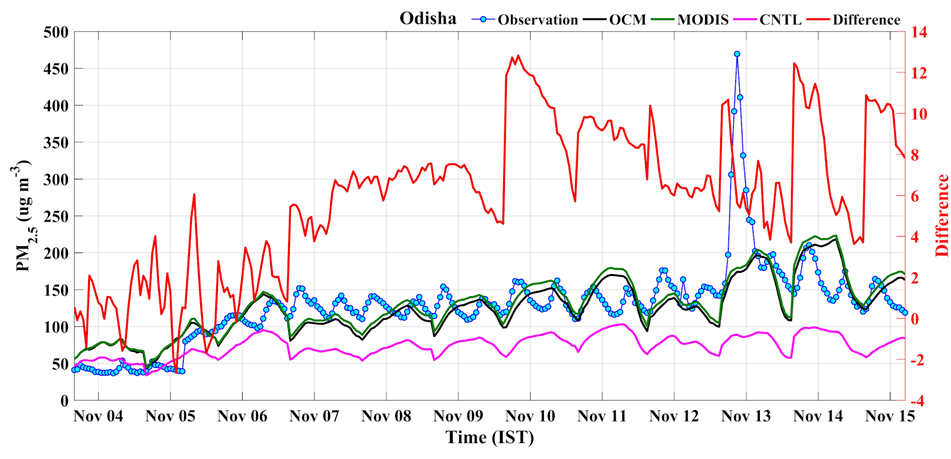


**(c)**


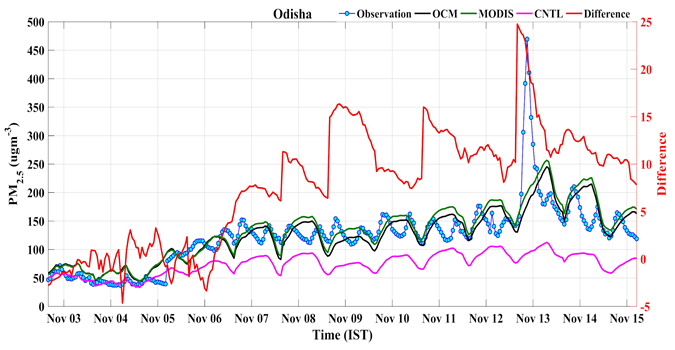


**(b)**

Figure S10. Time series of observed and WRF-Chem forecasted hourly PM₂.₅ mass concentrations in Odisha for the (a) first, (b) second, and (c) third forecast days. The left Y-axis represents PM₂.₅ concentrations (µg m⁻³), while the right Y-axis indicates the absolute difference between MODISDA and OCMDA forecasts. The line plots show observations (blue), CNTL (pink), MODIS (MODISDA, green), and OCM (OCMDA, black). The red line represents the MODISDA–OCMDA difference.

**(a)**


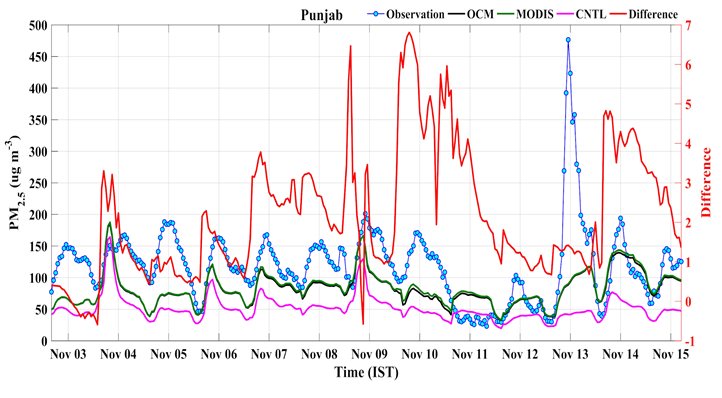


**(b)**


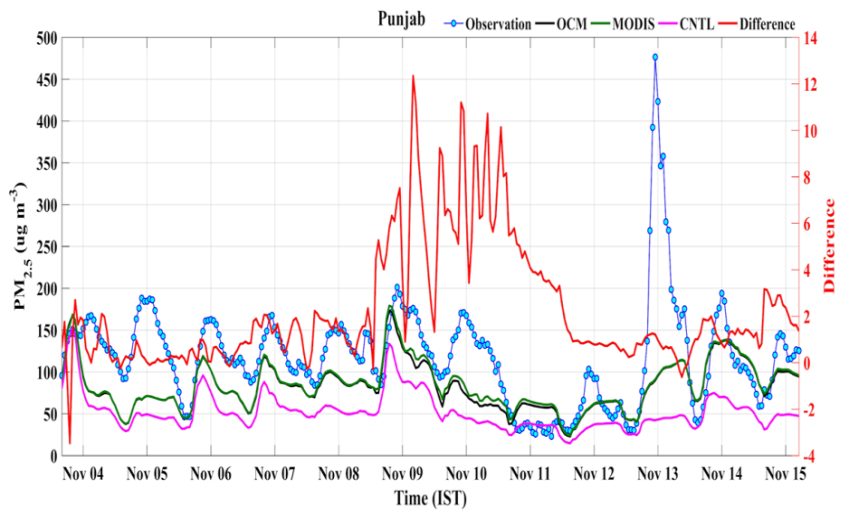


**(c)**


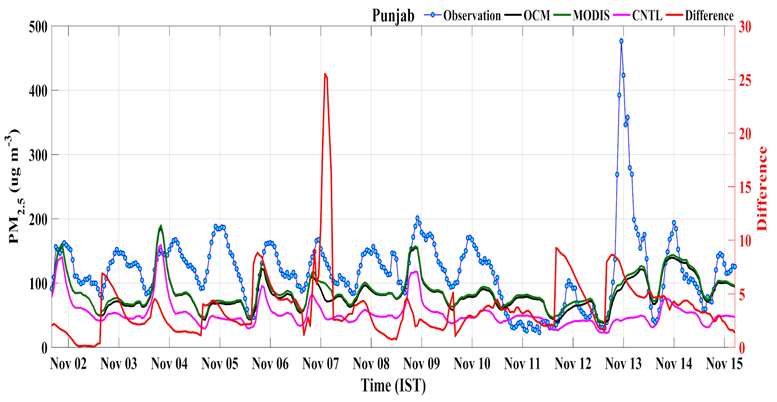


**(a)**

Figure S11. Time series of observed and WRF-Chem forecasted hourly PM₂.₅ mass concentrations in Punjab for the (a) first, (b) second, and (c) third forecast days. The left Y-axis represents PM₂.₅ concentrations (µg m⁻³), while the right Y-axis indicates the absolute difference between MODISDA and OCMDA forecasts. The line plots show observations (blue), CNTL (pink), MODIS (MODISDA, green), and OCM (OCMDA, black). The red line represents the MODISDA–OCMDA difference.

**(a)**


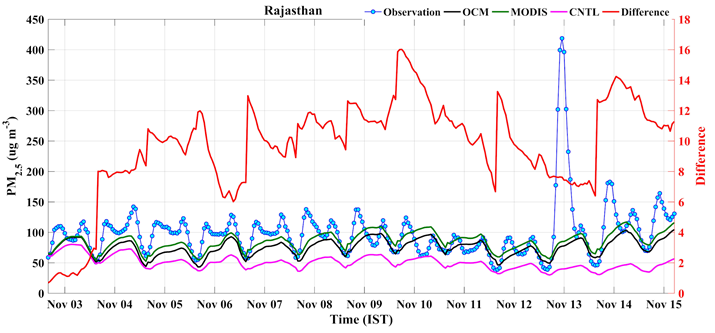


**(b)**


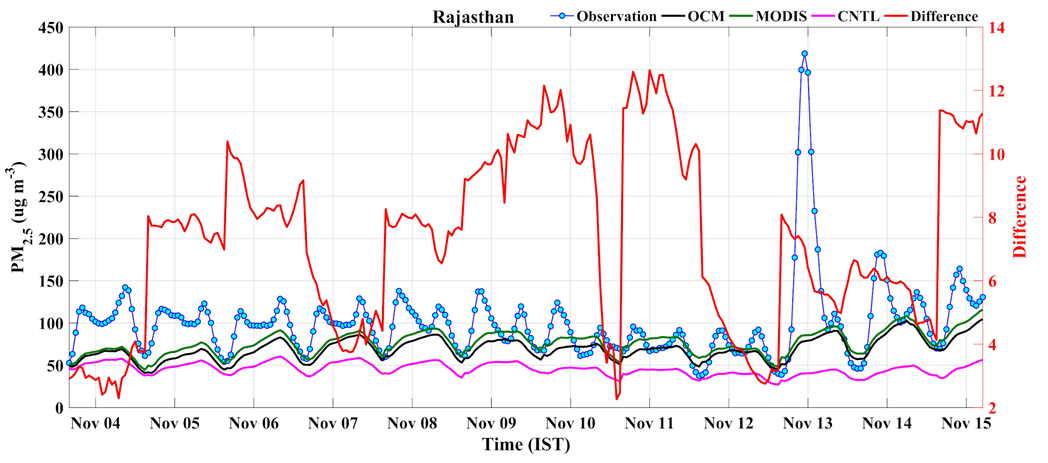


**(c)**


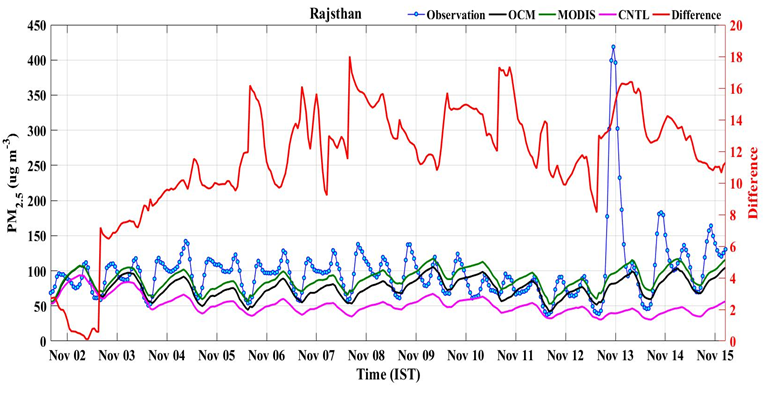


**(a)**

Figure S11. Time series of observed and WRF-Chem forecasted hourly PM₂.₅ mass concentrations in Rajasthan for the (a) first, (b) second, and (c) third forecast days. The left Y-axis represents PM₂.₅ concentrations (µg m⁻³), while the right Y-axis indicates the absolute difference between MODISDA and OCMDA forecasts. The line plots show observations (blue), CNTL (pink), MODIS (MODISDA, green), and OCM (OCMDA, black). The red line represents the MODISDA–OCMDA difference.


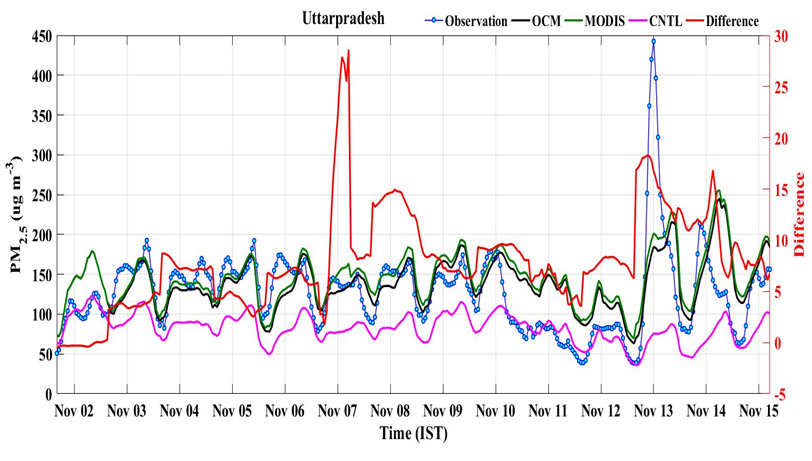

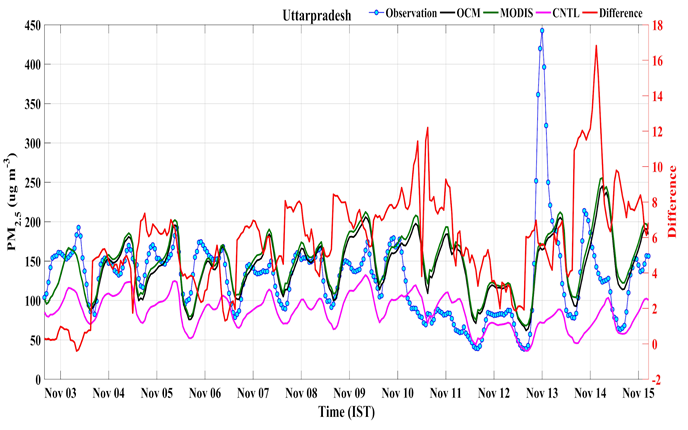

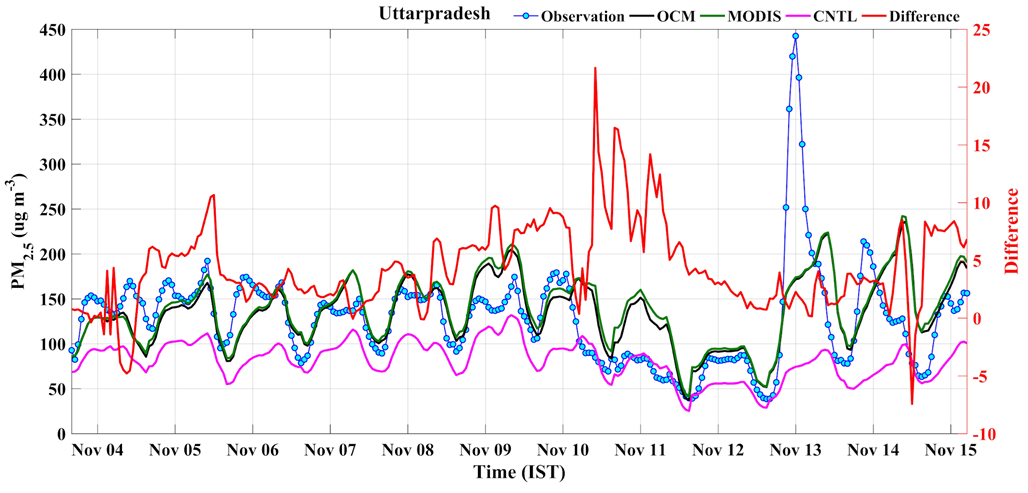


**(a)**

**(b)**

s

**(c)**

Figure S12. Time series of observed and WRF-Chem forecasted hourly PM₂.₅ mass concentrations in Uttarpradesh for the (a) first, (b) second, and (c) third forecast days. The left Y-axis represents PM₂.₅ concentrations (µg m⁻³), while the right Y-axis indicates the absolute difference between MODISDA and OCMDA forecasts. The line plots show observations (blue), CNTL (pink), MODIS (MODISDA, green), and OCM (OCMDA, black). The red line represents the MODISDA–OCMDA difference.


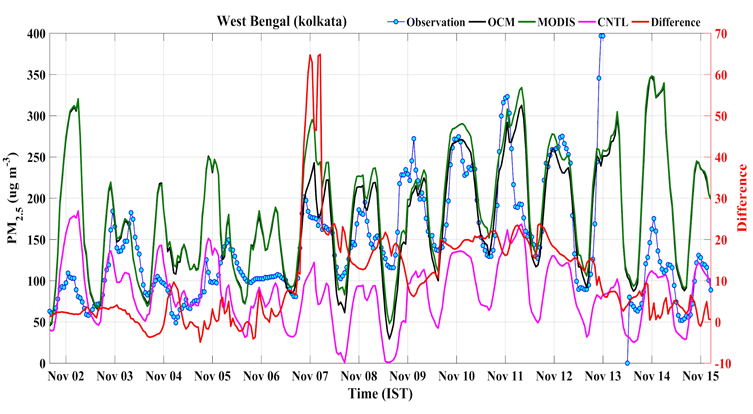


**(a)**


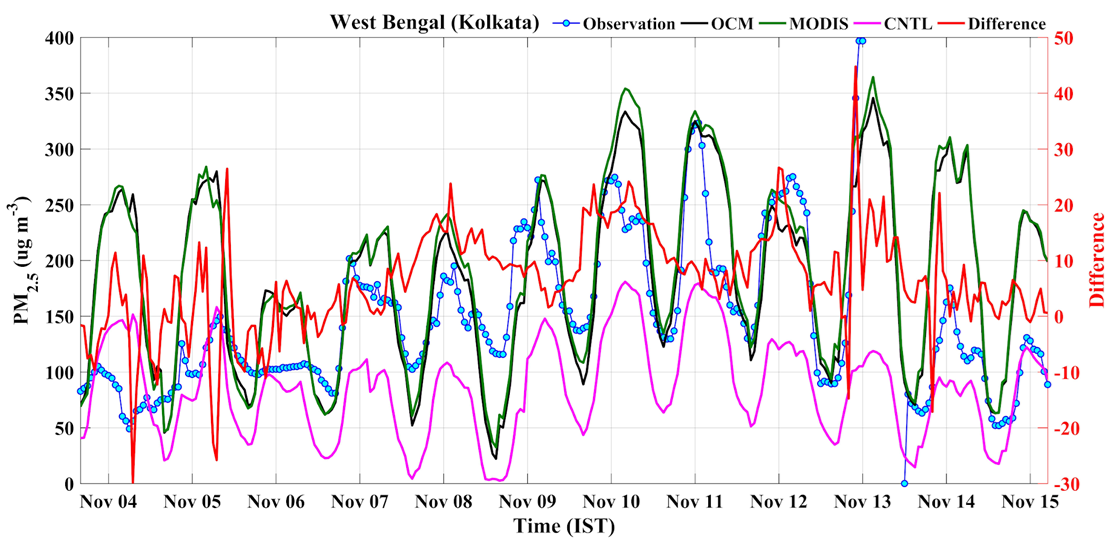


**(c)**


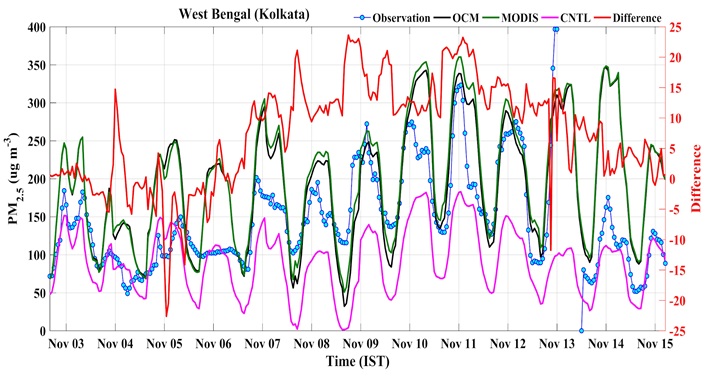


**(b)**

**(b)**

Figure S13. Time series of observed and WRF-Chem forecasted hourly PM₂.₅ mass concentrations in West Bengal (Kolkata) for the (a) first, (b) second, and (c) third forecast days. The left Y-axis represents PM₂.₅ concentrations (µg m⁻³), while the right Y-axis indicates the absolute difference between MODISDA and OCMDA forecasts. The line plots show observations (blue), CNTL (pink), MODIS (MODISDA, green), and OCM (OCMDA, black). The red line represents the MODISDA–OCMDA difference.

S2. **WRF-Chem Model Configuration**

In this study, we used the Weather Research and Forecasting model coupled with Chemistry (WRF-Chem, v3.9.1. The outermost domain (d01) covers the Indian subcontinent at a horizontal resolution of **10 km × 10 km**. The present analysis uses results from the **10 km resolution** domain to evaluate regional-scale impacts of OCM AOD assimilation.

Meteorological initial and boundary conditions are taken from the IITM-GFS (T1534) spectral model with 12.5 km resolution, updated every 3 h. Chemical boundary conditions for d01 are based on the 10-year climatology of MOZART-4, updated every 6 h, with outputs from outer domains providing nested boundary conditions for inner domains.

Physics and chemistry parameterizations**:**

- **Microphysics:** Morrison double-moment scheme^1^ to represent cloud–aerosol interactions.
- **Radiation:** Rapid Radiative Transfer Model for GCMs (RRTMG^2^) for both shortwave and longwave processes, including aerosol–radiation feedbacks.
- **Cumulus parameterization:** Grell–Freitas scheme^3^ applied only in d01.
- **Land surface:** Noah land surface model (Tewari et al., 2004).
- **Planetary boundary layer:** Mellor–Yamada–Nakanishi–Niino Level 2.5^5^.
- **Surface layer:** Monin–Obukhov (Janjic Eta) scheme.
- **Aerosol and chemistry:** MOZART-4 gas-phase chemistry coupled with the GOCART aerosol module^7,8^ for sulfate, black carbon, organic carbon, dust, and sea salt.

**Emissions:**

- Anthropogenic emissions were taken from the EDGAR-HTAP v2.2 global inventory (2010 base year) and scaled to the study period using the sector-specific growth factors of Venkataraman et al. (2018)^10^. To better represent local sources, these were merged with the high-resolution SAFAR Delhi inventory (400 m × 400 m, 2018; SAFAR-Delhi-2018^11^), replacing EDGAR-HTAP values within the Delhi domain.
- Biogenic emissions calculated online using MEGAN^12^.
- Open biomass burning from FINN^13^
- Diurnal emission profiles from Govardhan et al. (2019)^14^.

**S3. GSI 3DVAR data-assimilation configuration**

1. System and control variables

We assimilate satellite AOD using the community GSI (v3.5) three-dimensional variational (3DVAR) scheme coupled to WRF-Chem/GOCART. The state vector contains GOCART aerosol species (sulfate, hydrophilic/hydrophobic OC and BC, dust bins, sea-salt bins) and the meteorological variables required by the AOD forward model. The cost function follows the standard near-quadratic form, with AOD simulated by the Community Radiative Transfer Model (CRTM) as the observation operator, and minimization performed with the preconditioned conjugate-gradient solver (tolerance 10⁻⁹; max 50 iterations; typical 10–45).

1. Background-error statistics and localization.

Background error covariance (BEC) parameters are computed off-line with GEN_BE using the NMC method (differences of two 24-h forecasts valid at the same time). Resulting horizontal and vertical length scales are ~1–3 grid intervals, with largest variances in the boundary layer (<3 km), consistent with greater near-surface aerosol uncertainty. These statistics control the amplitude of analysis increments and their horizontal/vertical spread.

1. Analysis time, windowing, and cycling

We run a single daily analysis at 09:00 UTC, aligned with the MODIS Aqua overpass; near-real-time MODIS AOD is assumed available at this analysis time (NRT latency ≈3 h). GSI’s 3DVAR treats the analysis as instantaneous (no explicit 4-D time window); observations are collocated to the analysis time. Each analysis initializes a 72-h forecast.

1. Vertical grid

The forecasts use 50 sigma-levels with the model top at 50 hPa; DA increments are applied on all levels, with vertical influence constrained by the BEC length scales noted above.

1. Observation operator and errors

CRTM converts model aerosols to AOD at the satellite wavelength; hygroscopic growth and aerosol optical properties follow standard look-up tables as in prior WRF-Chem/GSI AOD DA studies.


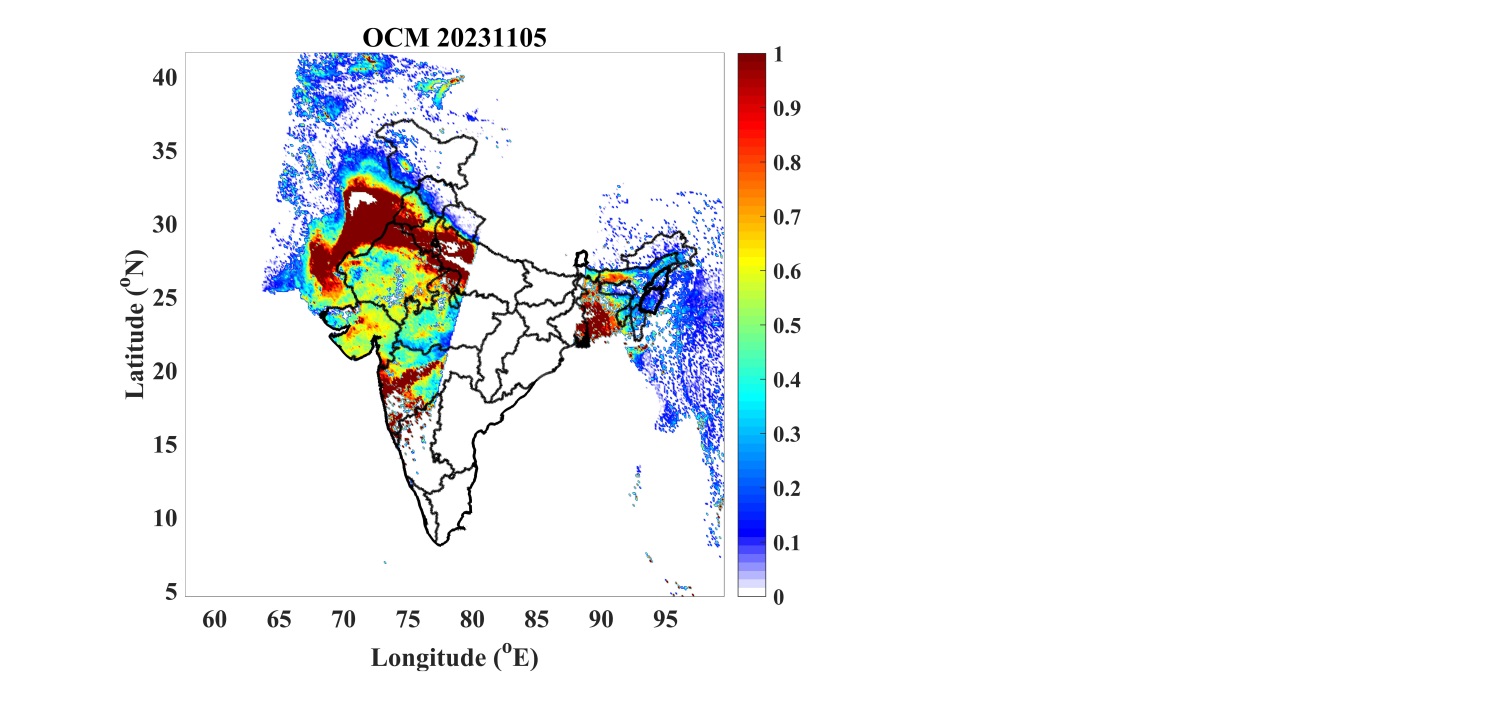


(a)


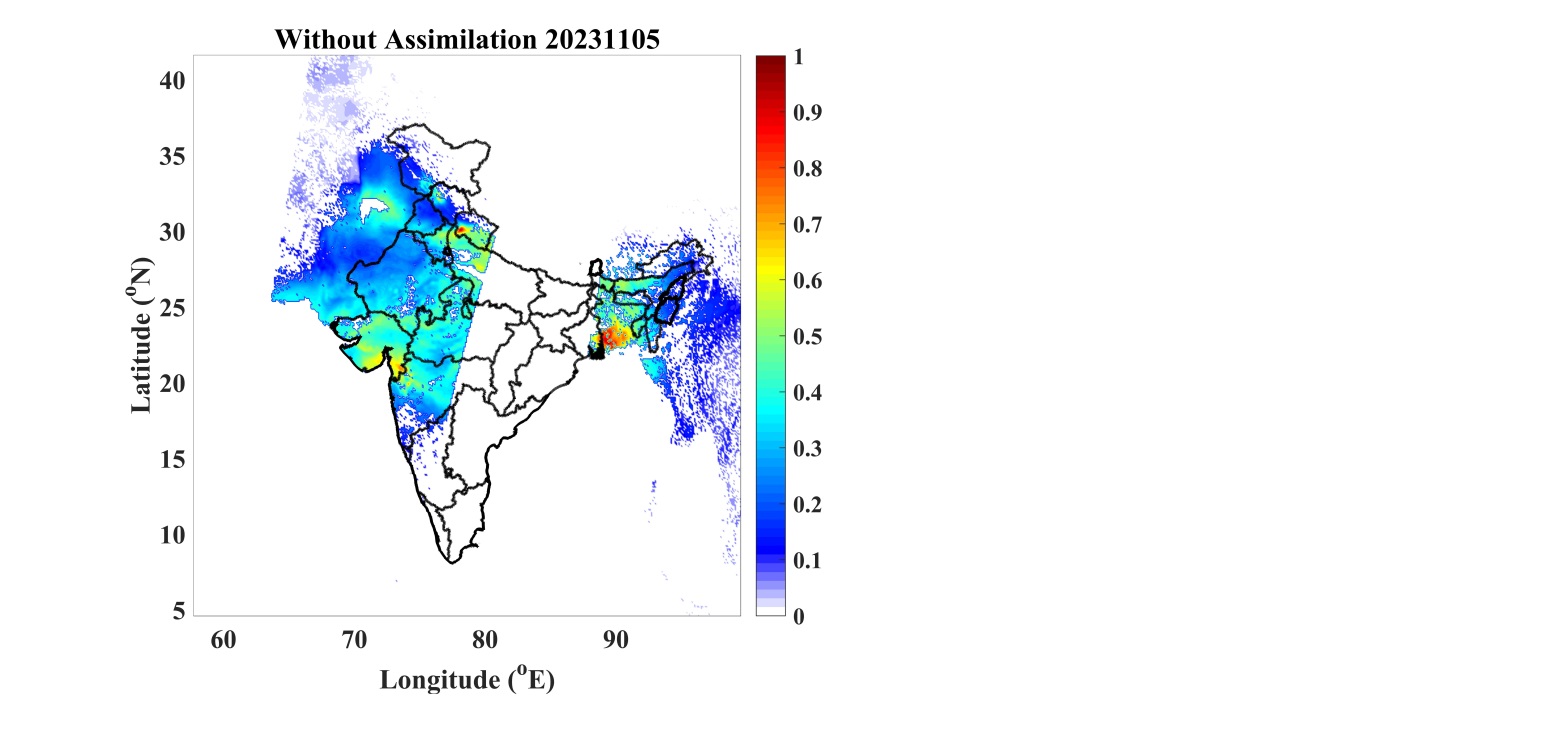


(b)


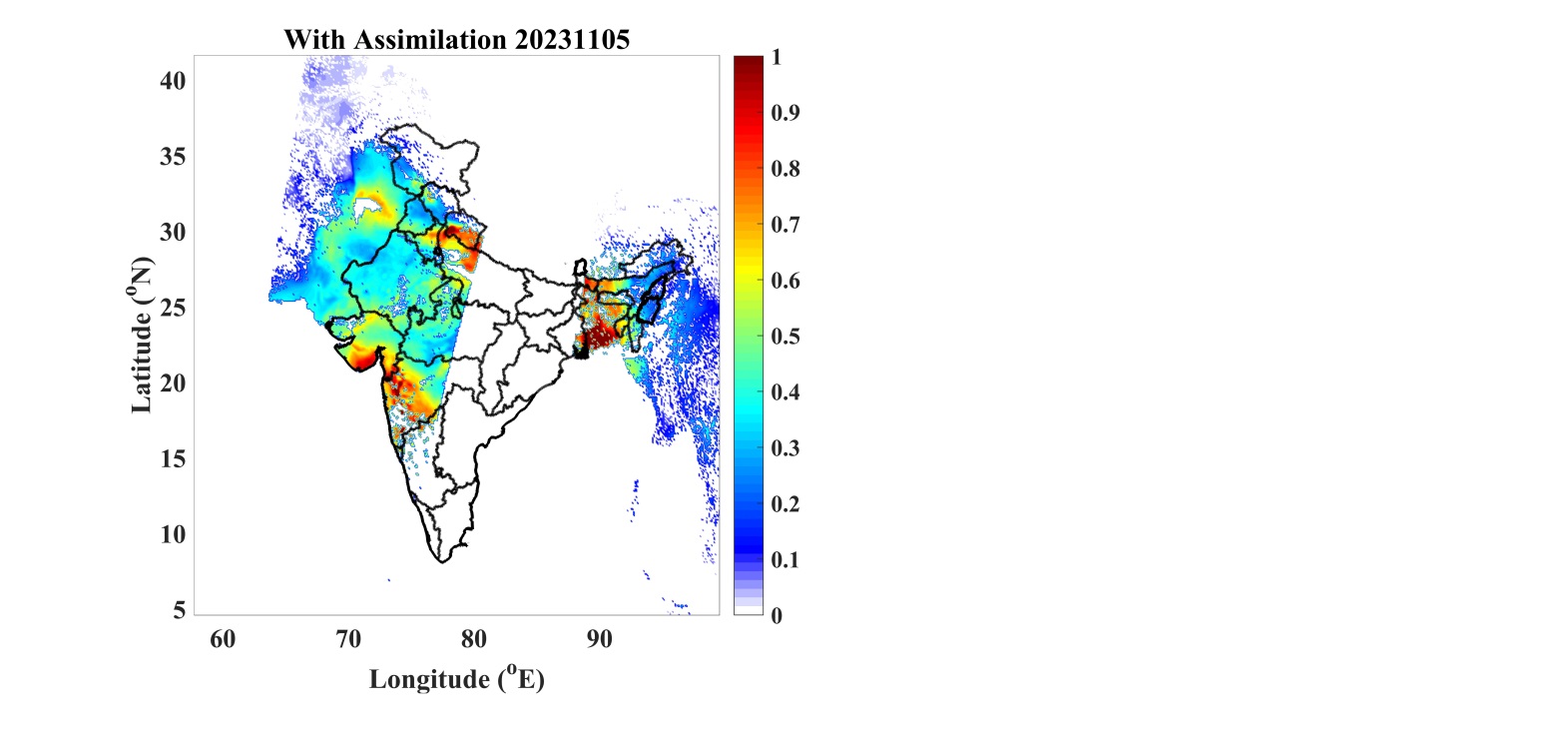


(c)


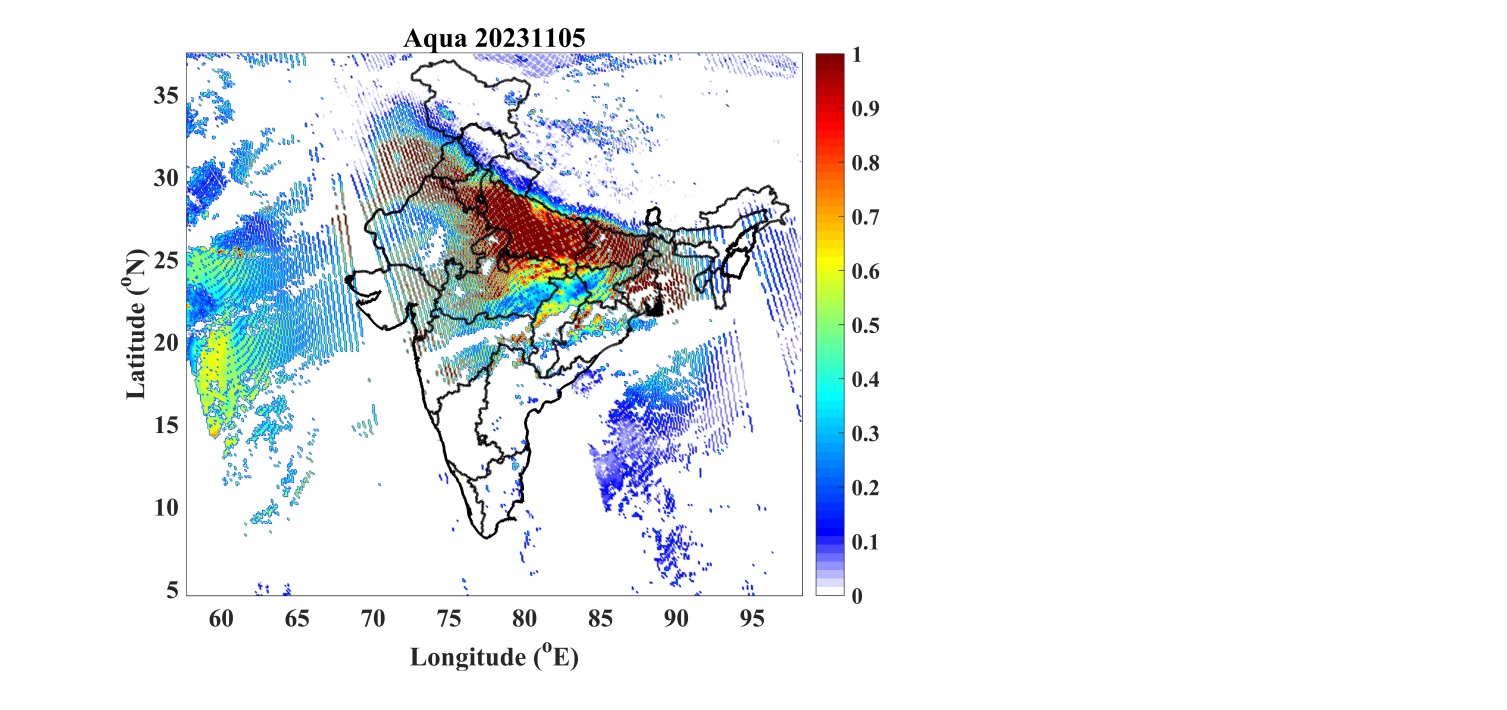


(d)


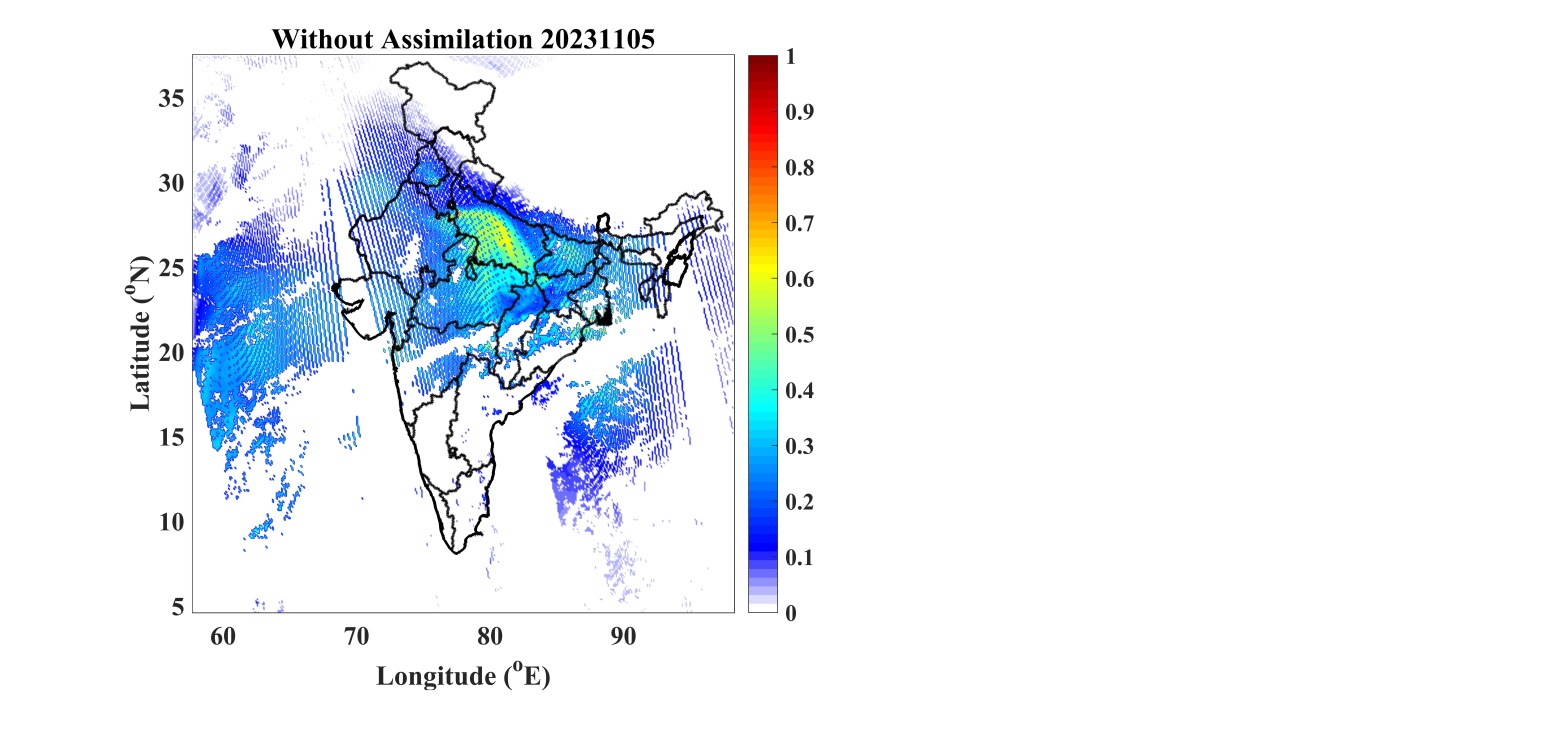


(e)


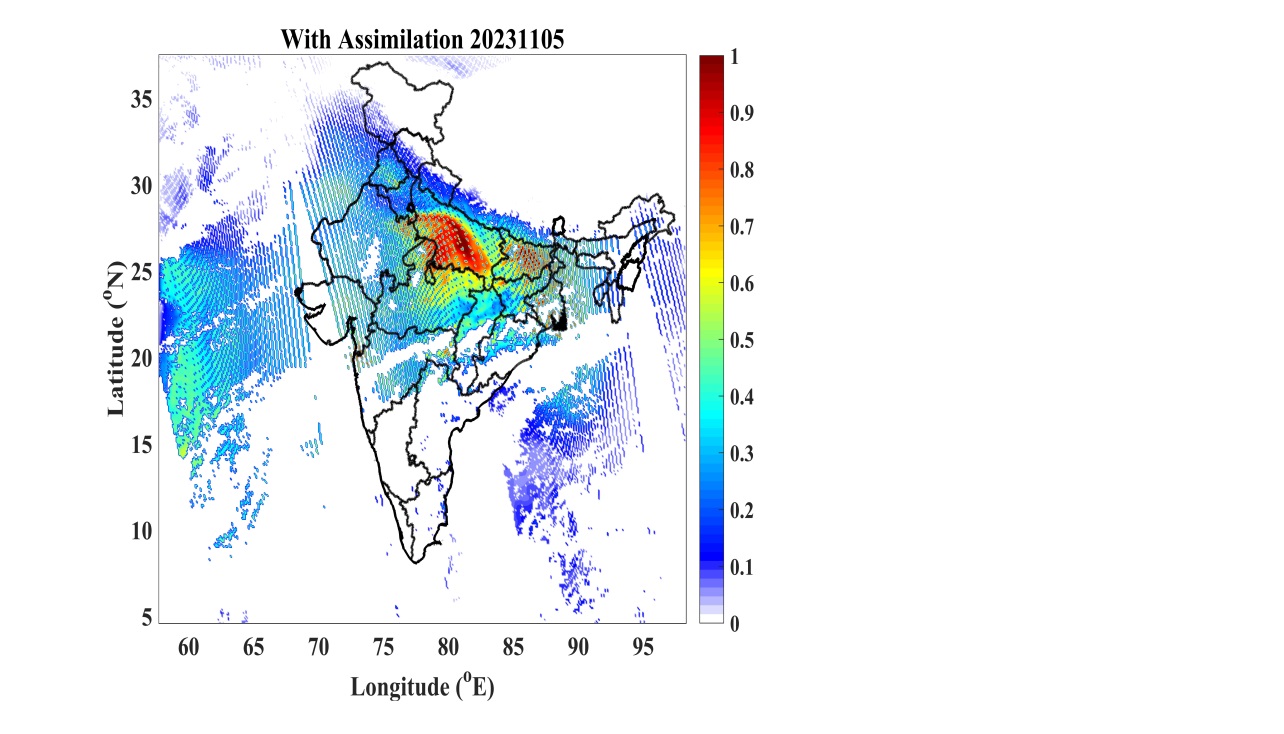


(f)


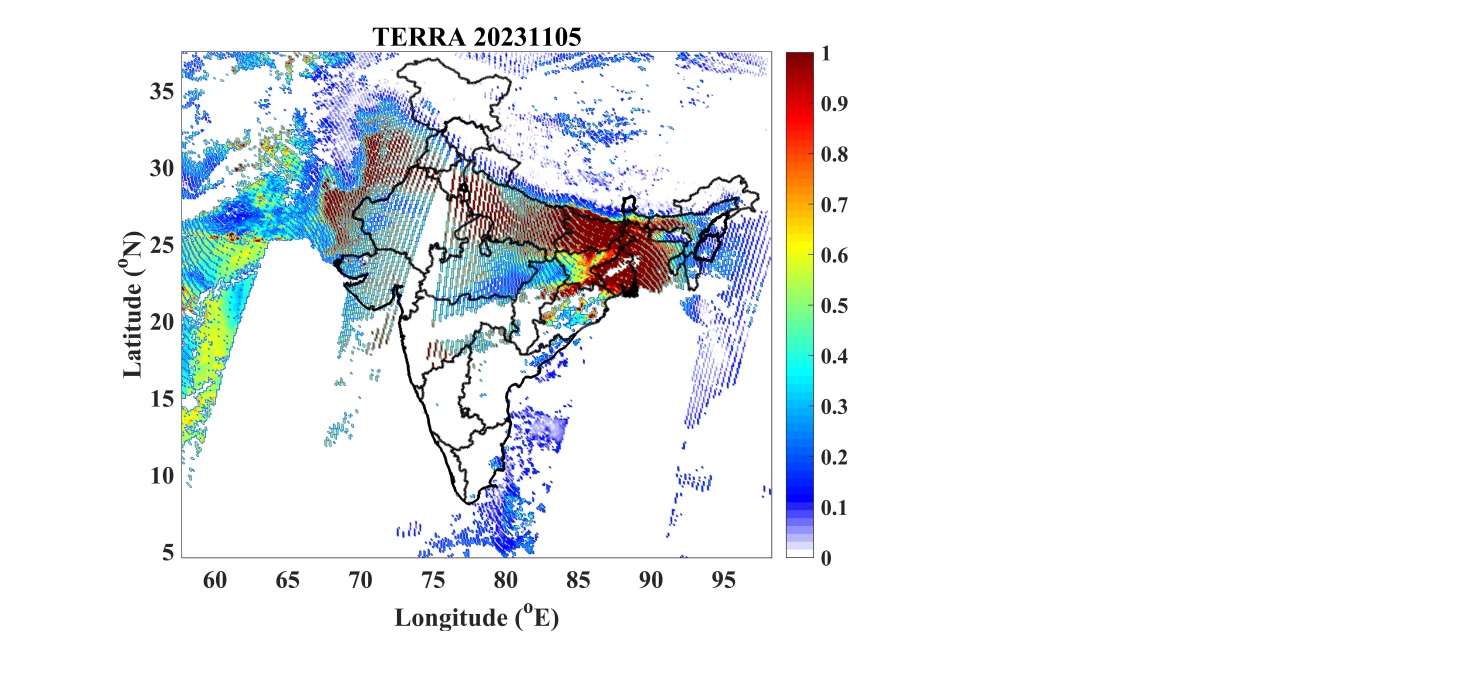


(g)


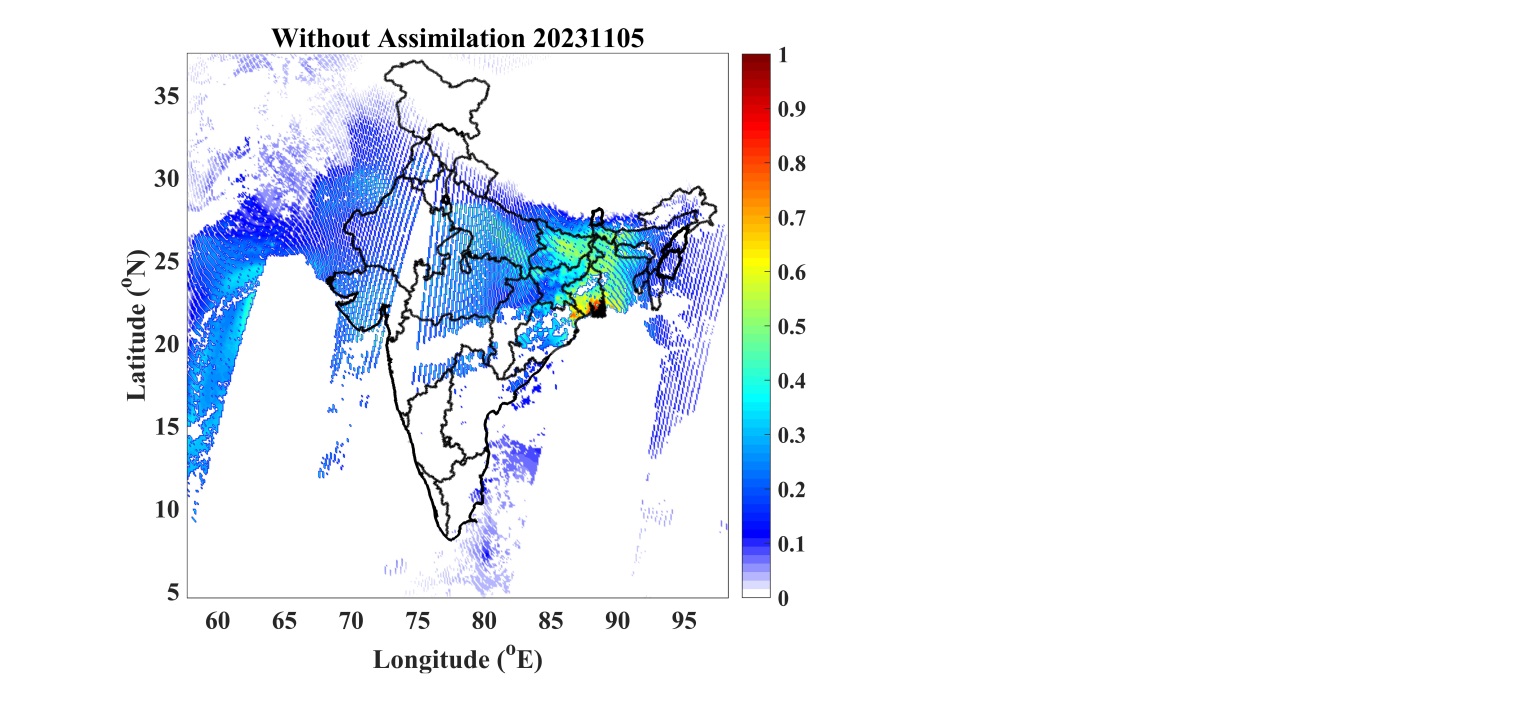


(h)


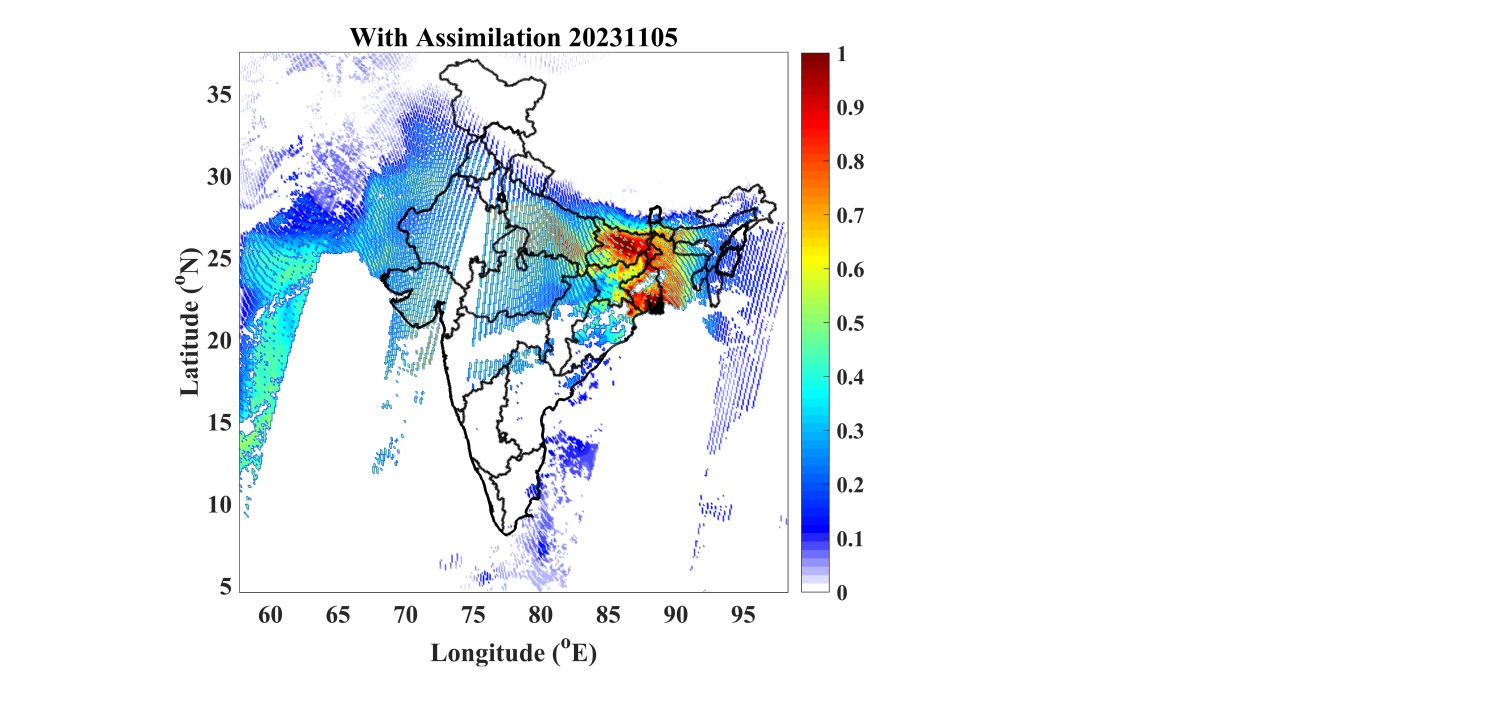


(i)

Figure S14. Spatial distribution of AOD at 550 nm on 05 November 2023 at 09:00 UTC, comparing observations and model simulations. The first column shows observed AOD from OCM, Aqua MODIS, and Terra MODIS satellite swaths (panels a, d, g). The second column presents WRF-Chem simulations without data assimilation (CNTL experiment; panels b, e, h),.The third column displays simulations after assimilating satellite AOD: OCM AOD assimilation (OCMDA; panel c) and MODIS AOD assimilation (MODISDA; panels f, i).


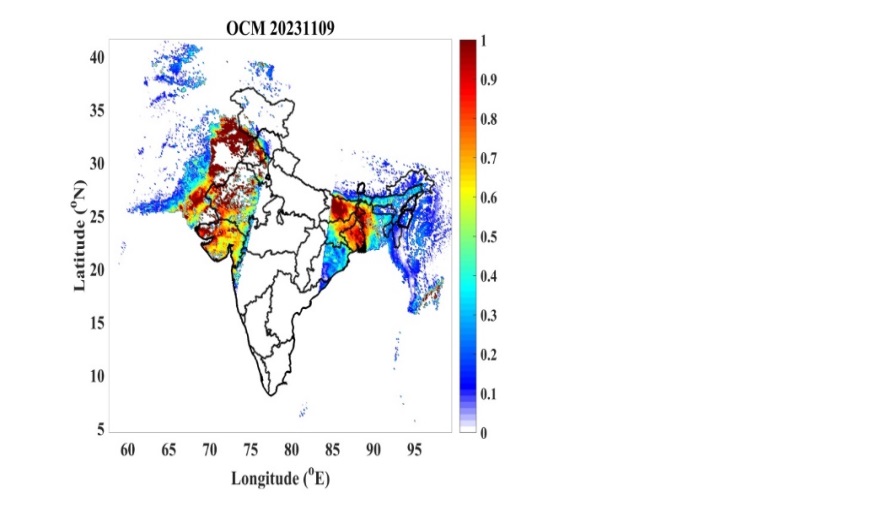


(a)


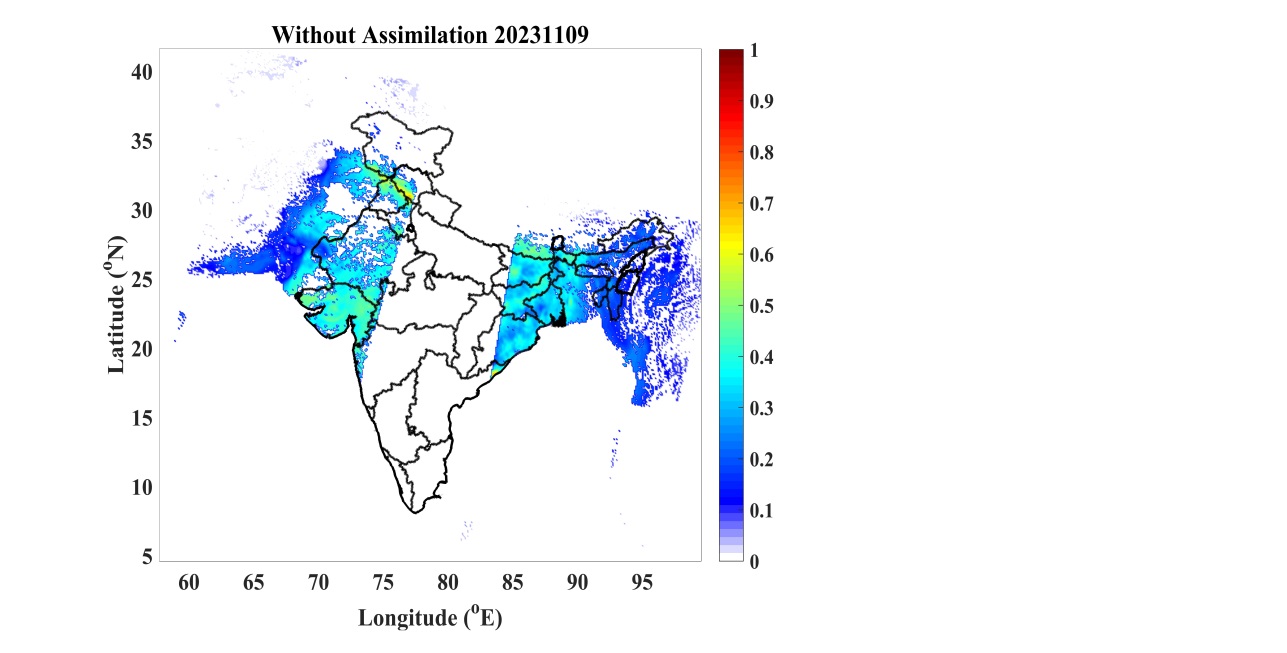


(b)


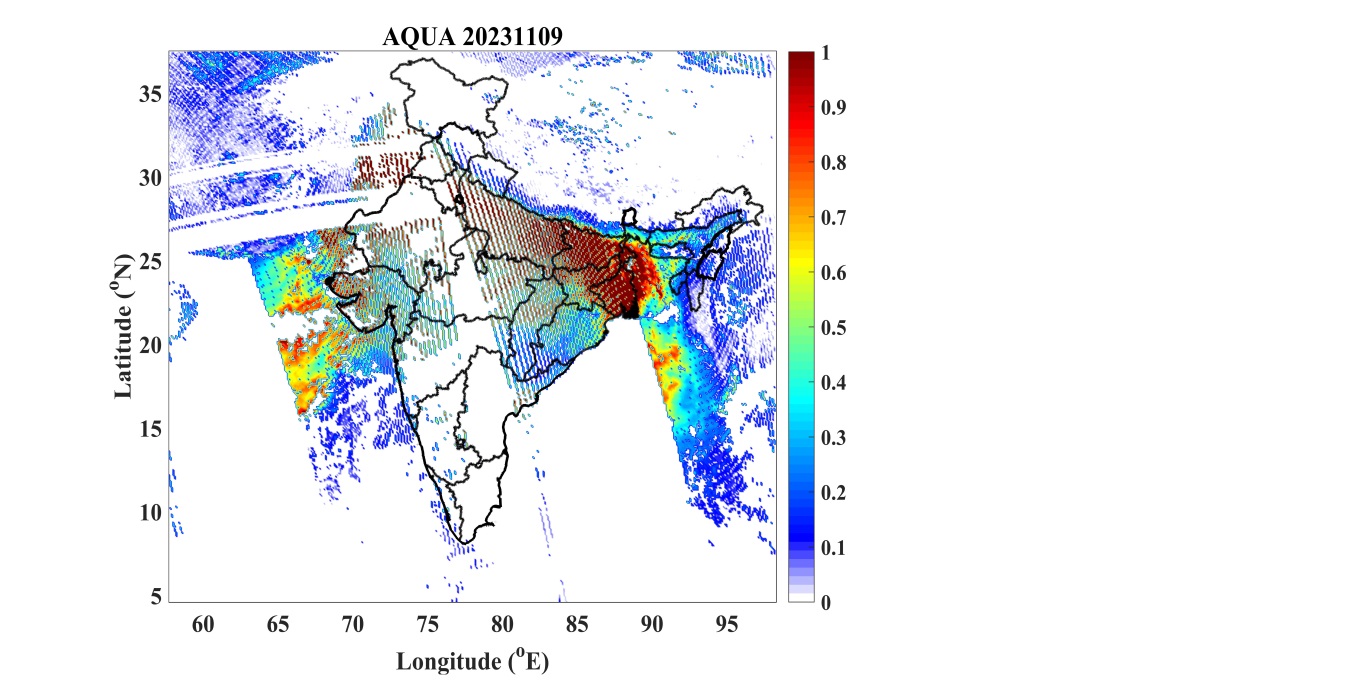


(d)


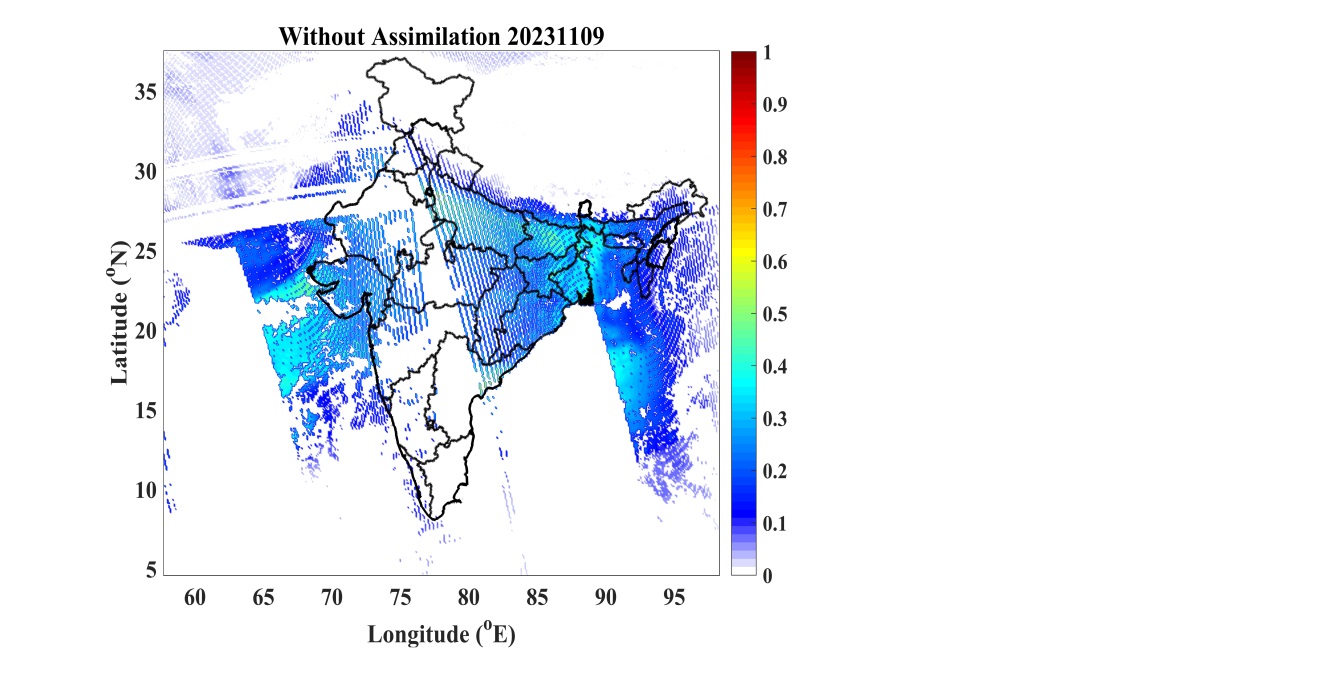


(e)


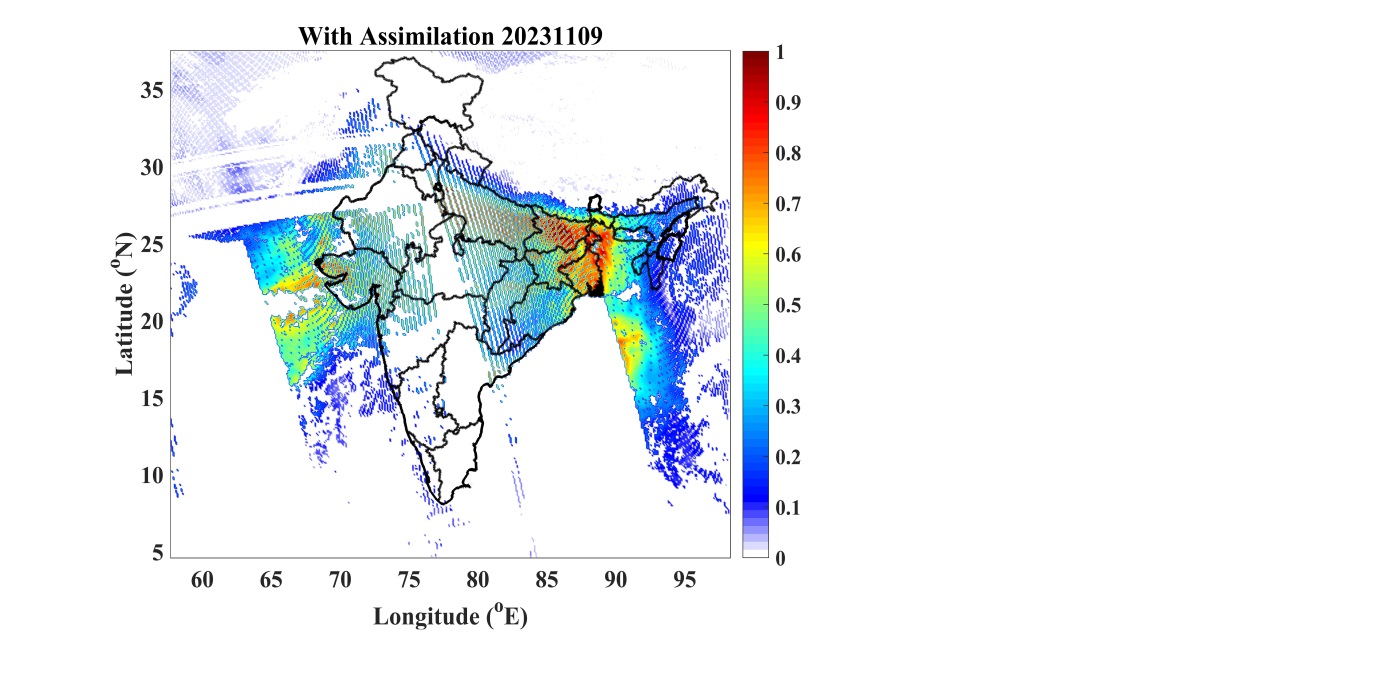


(f)


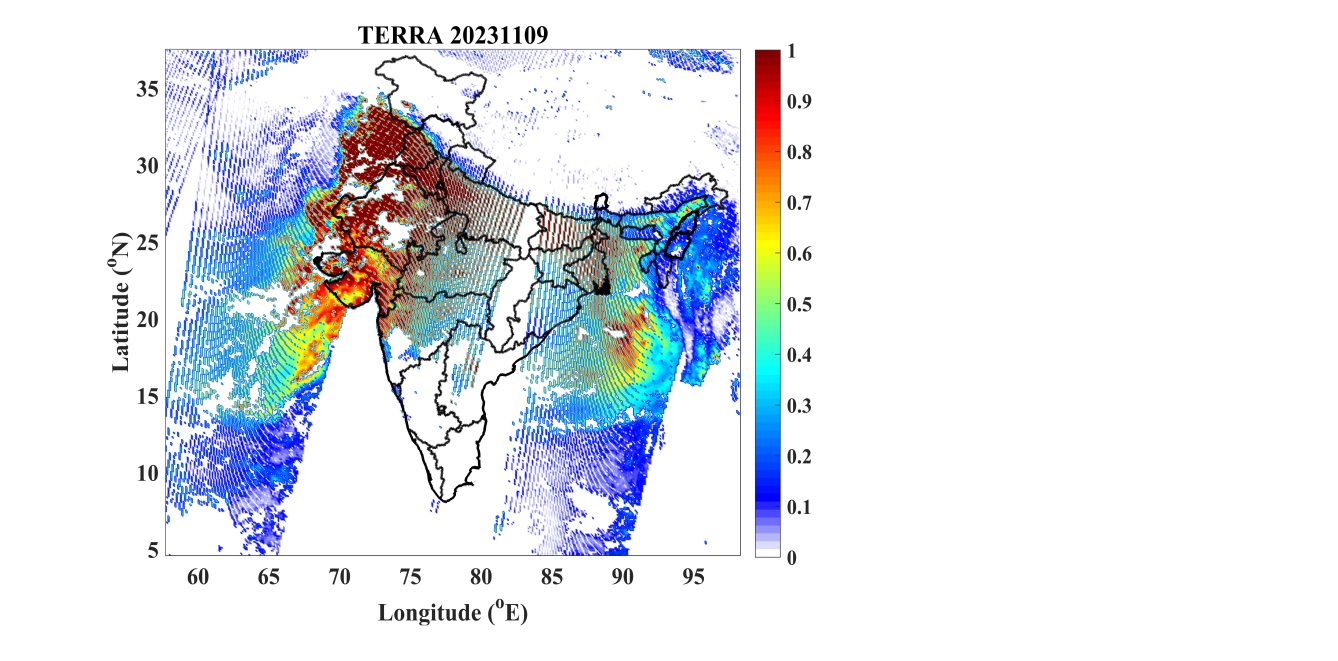


(g)


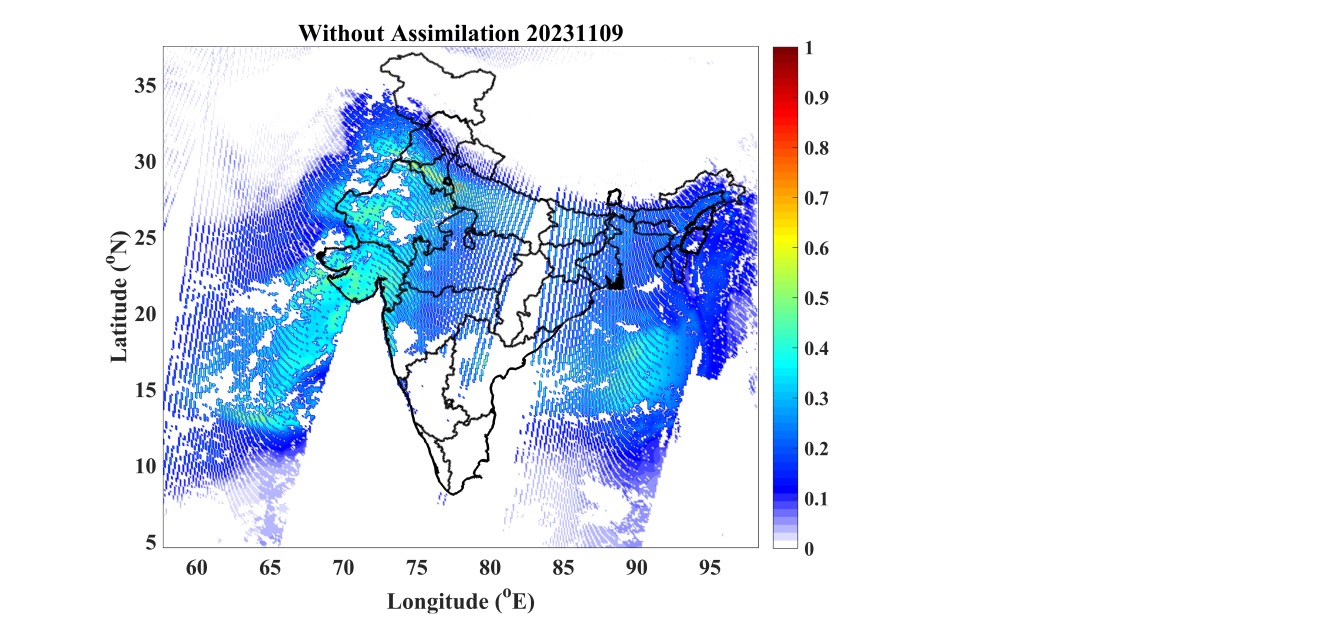


(h)


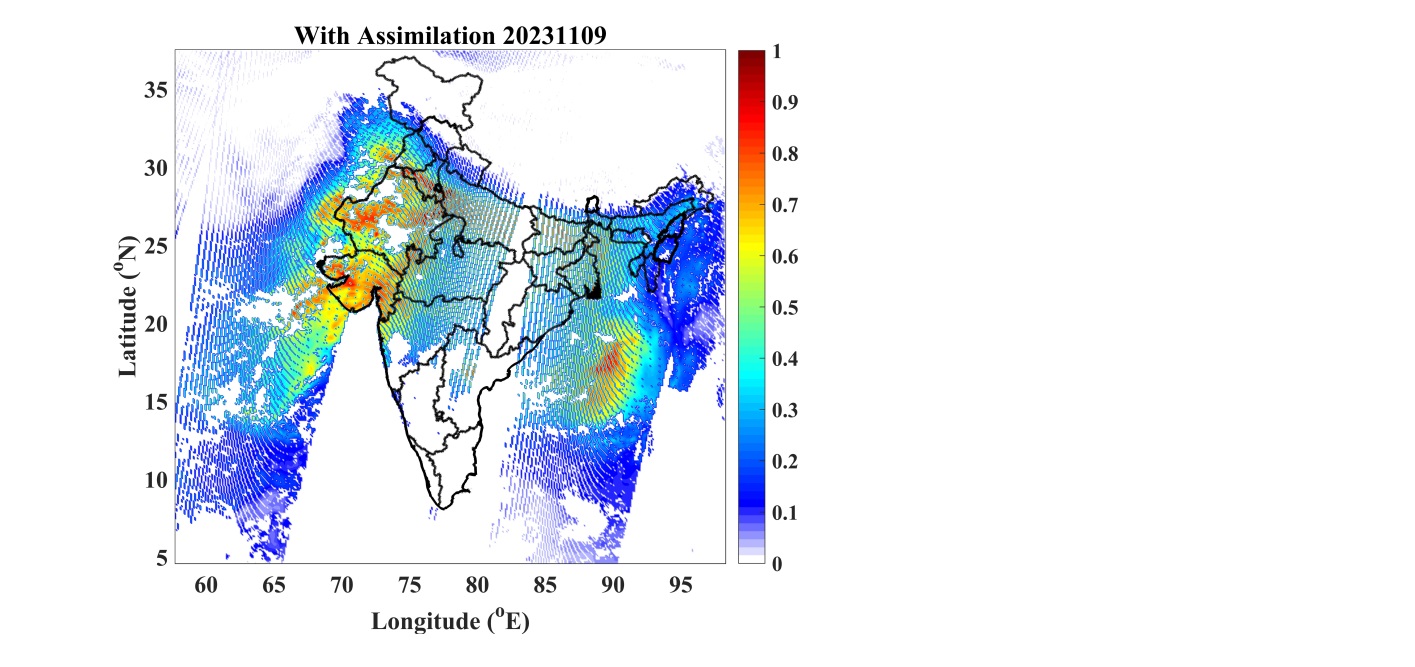


(i)


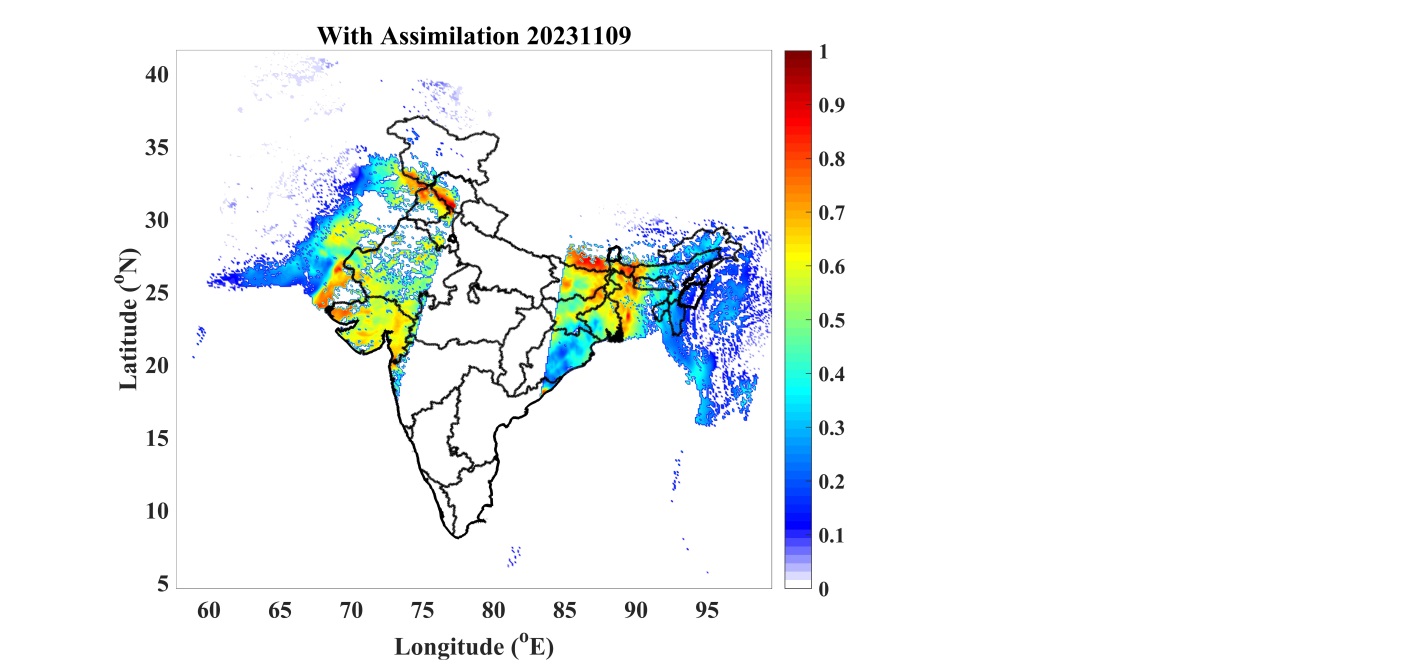


(c)

Figure S15. Spatial distribution of AOD at 550 nm on 09 November 2023 at 09:00 UTC, comparing observations and model simulations. The first column shows observed AOD from OCM, Aqua MODIS, and Terra MODIS satellite swaths (panels a, d, g). The second column presents WRF-Chem simulations without data assimilation (CNTL experiment; panels b, e, h),.The third column displays simulations after assimilating satellite AOD: OCM AOD assimilation (OCMDA; panel c) and MODIS AOD assimilation (MODISDA; panels f, i).


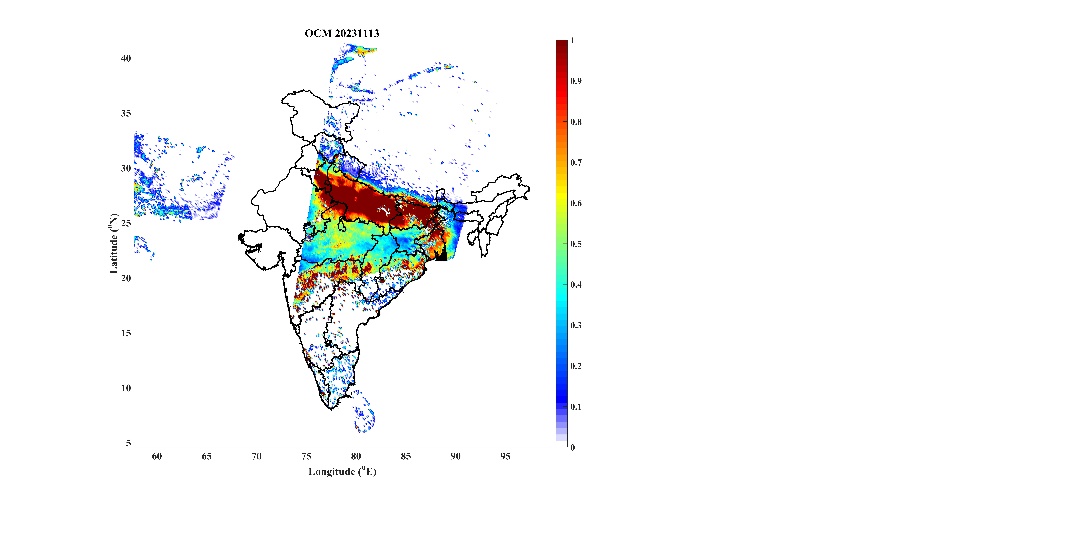


(a)


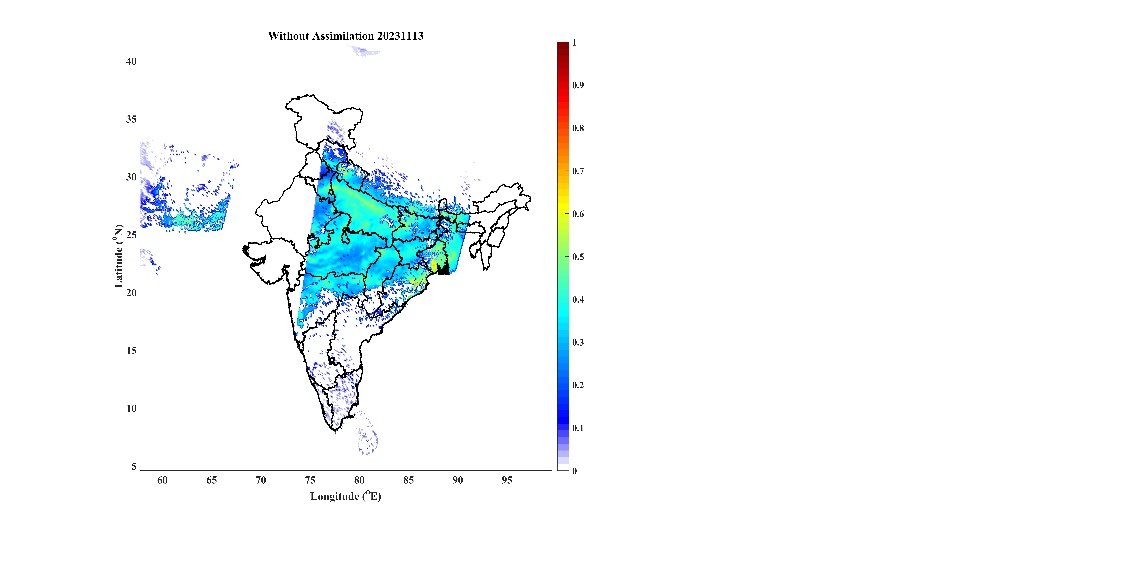


(b)


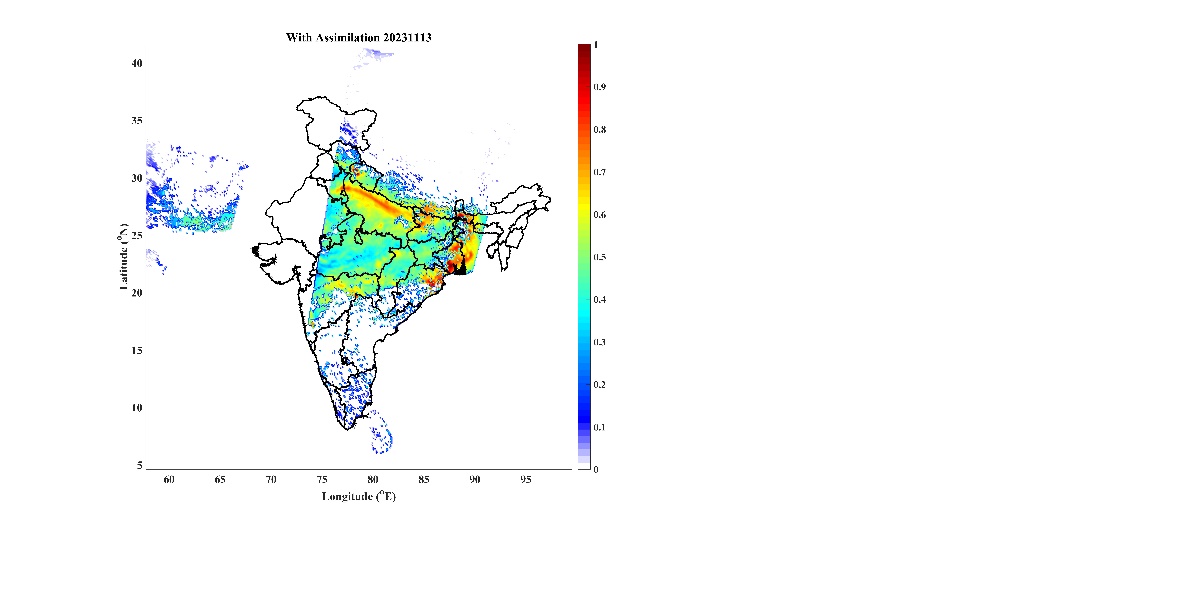


(c)


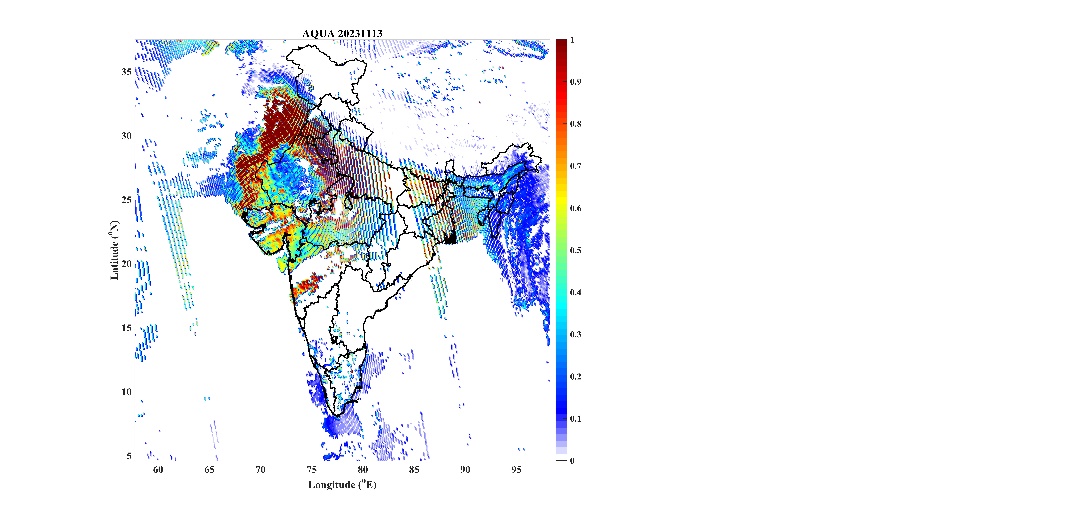


(d)


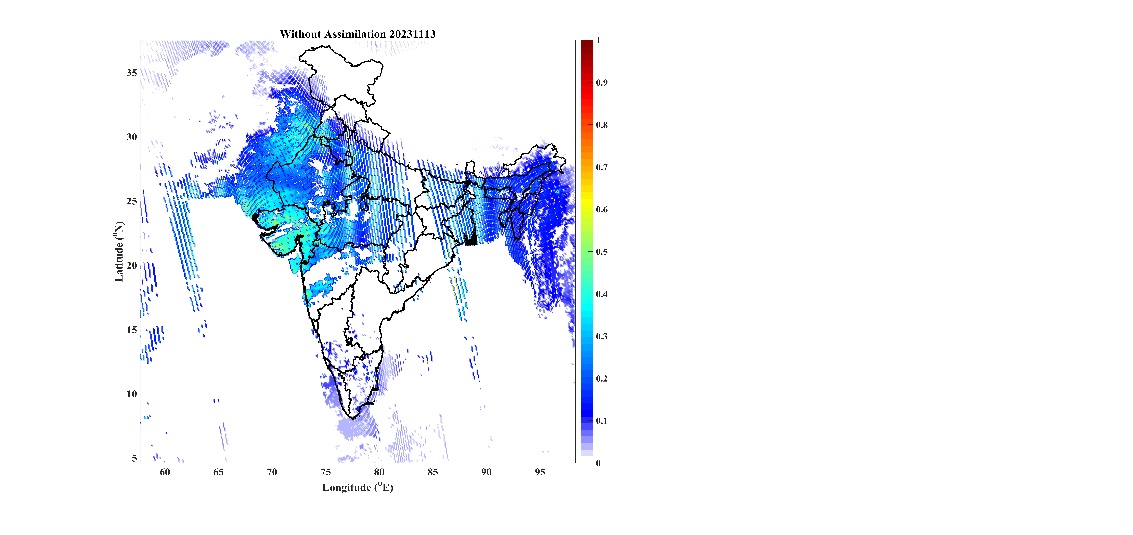


(e)


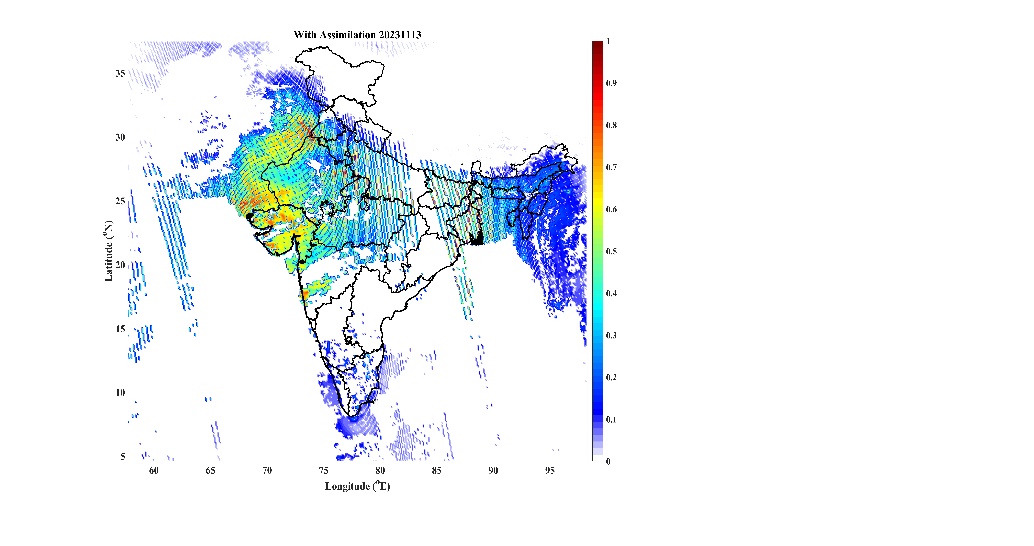


(f)


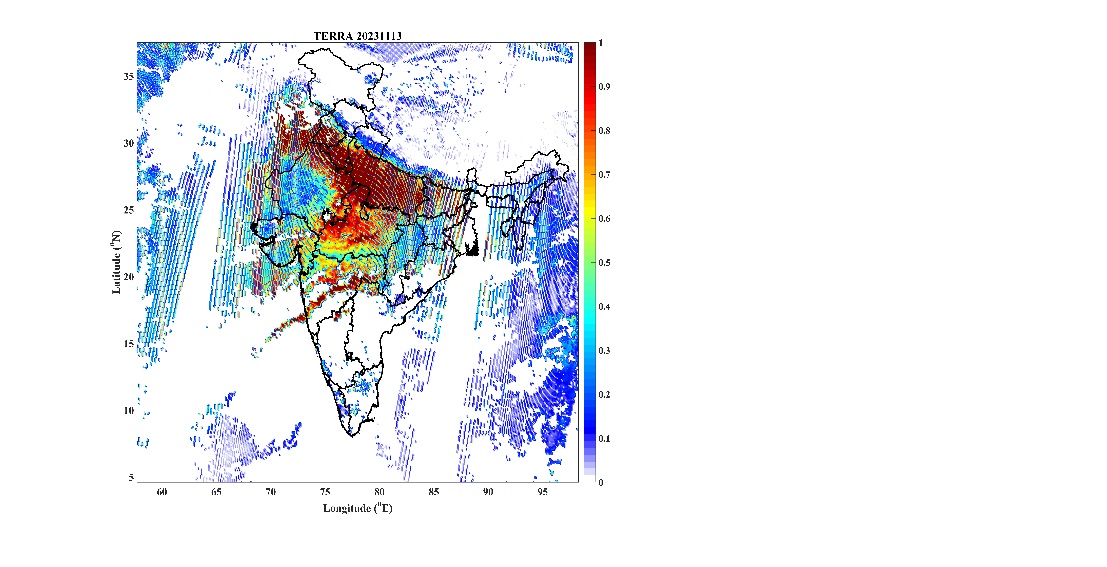


(g)


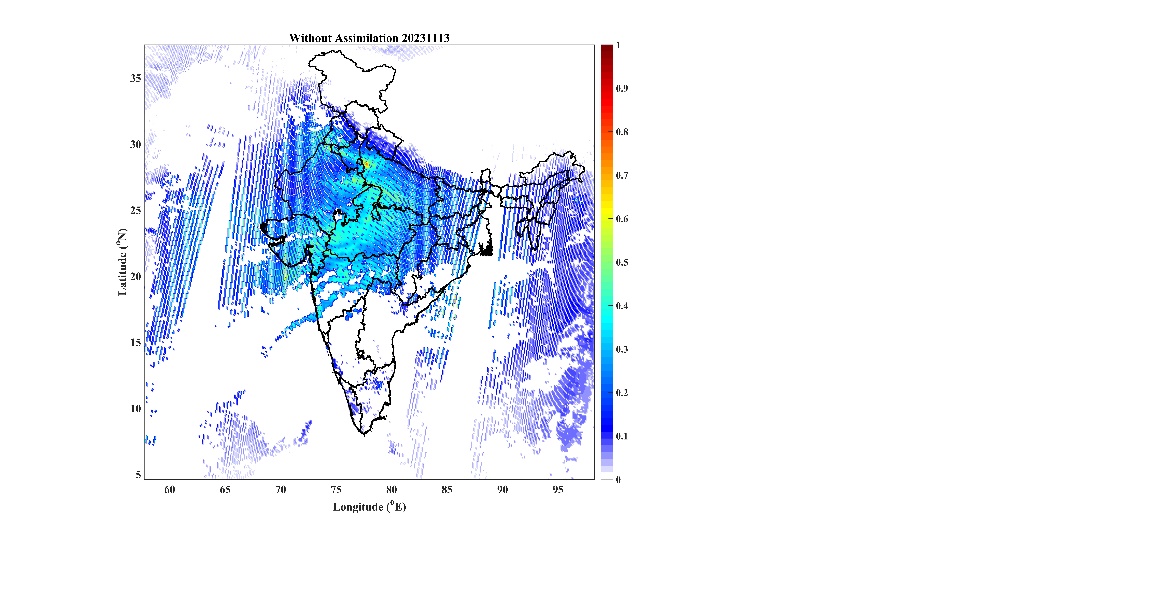


(h)


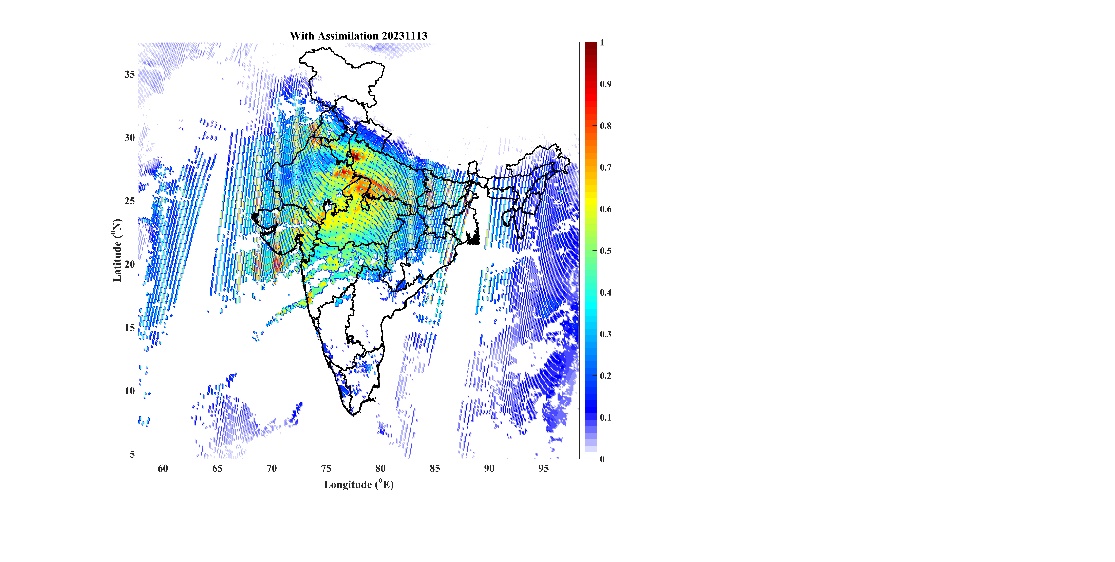


(i)

Figure S15. Spatial distribution of AOD at 550 nm on 13 November 2023 at 09:00 UTC, comparing observations and model simulations. The first column shows observed AOD from OCM, Aqua MODIS, and Terra MODIS satellite swaths (panels a, d, g). The second column presents WRF-Chem simulations without data assimilation (CNTL experiment; panels b, e, h),.The third column displays simulations after assimilating satellite AOD: OCM AOD assimilation (OCMDA; panel c) and MODIS AOD assimilation (MODISDA; panels f, i).

On 05 November 2023, satellite observations from OCM, Aqua MODIS, and Terra MODIS revealed widespread high AOD values exceeding 1.0 over Punjab and Haryana, extending into Delhi. The CNTL simulation underestimated aerosol loading across the Indo-Gangetic Plain, particularly in downwind regions affected by biomass burning. Assimilation of OCM and MODIS AOD significantly increased aerosol concentrations over both the source and receptor areas, reducing spatial bias and improving agreement with observed plumes.

On 09 November 2023, AOD peaks were observed over central Punjab and northwestern Uttar Pradesh, with elevated values over Delhi. Similar to the earlier case, the CNTL run underestimated aerosol loading in the downwind regions. Both OCMDA and MODISDA captured the spatial extent and magnitude of the plume more realistically, with OCMDA showing slightly better agreement along plume edges, likely due to its higher spatial resolution.

On 13 November 2023, one of the season’s most severe PM₂.₅ episodes occurred over Delhi, driven by residual biomass burning and unfavourable meteorological conditions such as low ventilation and temperature inversion. Observed AOD remained above 0.9 across much of the Indo-Gangetic Plain. The CNTL simulation again underestimated aerosol concentrations over Delhi, whereas both assimilation runs reproduced the elevated load and its spatial distribution more accurately, with notable improvements in the urban core.

Overall, these additional cases confirm that data assimilation substantially improves the model’s ability to reproduce high-pollution episodes during the biomass burning period, enhancing both the magnitude and spatial representation of aerosol loading across multiple peak days.

Table S3. Daily number of assimilated AOD retrievals from MODIS (Terra + Aqua) and OCM during the study period (1–14 November 2023).

| **Sr.No.** | **Date** | **OCM** | **MODIS (Aqua+Terra)** |
| --- | --- | --- | --- |
| 1 | 2023/11/01 | 1121 | 1747 |
| 2 | 2023/11/02 | 1423 | 1046 |
| 3 | 2023/11/03 | 1391 | 1984 |
| 4 | 2023/11/04 | 1393 | 941 |
| 5 | 2023/11/05 | 1396 | 1880 |
| 6 | 2023/11/06 | 1421 | 1305 |
| 7 | 2023/11/07 | 1403 | 2198 |
| 8 | 2023/11/08 | 1210 | 1819 |
| 9 | 2023/11/09 | 1287 | 2023 |
| 10 | 2023/11/10 | 1160 | 2462 |
| 11 | 2023/11/11 | 1457 | 1402 |
| 12 | 2023/11/12 | 1324 | 2165 |
| 13 | 2023/11/13 | 1216 | 2018 |
| 14 | 2023/11/14 | 1405 | 1212 |

Table S4. Day-wise mean observed PM₂.₅ concentrations (µg m⁻³) for each state used in the evaluation of CNTL, MODISDA, and OCMDA experiments.

| **State** | **Observed PM_2.5_ (ug m^3^) Day1** | **Observed PM_2.5_ (ug m^3^) Day2** | **Observed PM_2.5_ (ug m^3^) Day3** |
| --- | --- | --- | --- |
| Bihar | 129.9 | 131.7 | 132.6 |
| Delhi | 256.9 | 258 | 248.1 |
| Gujarat | 95.3 | 94.9 | 94.8 |
| Haryana | 169.3 | 169 | 164.8 |
| Jharkhand | 149.1 | 141.8 | 140.3 |
| Maharashtra (Pune) | 74.3 | 72.2 | 71.1 |
| Madhya Pradesh | 91.6 | 92.3 | 93.6 |
| Odisha | 123.4 | 128.7 | 136.1 |
| Punjab | 118.4 | 118.3 | 118.1 |
| Rajasthan | 97.6 | 98.7 | 99.4 |
| Uttar pradesh | 127 | 129.1 | 127.6 |
| West Bengal (Kolkata) | 159.7 | 161.6 | 164.9 |
| Chattisgarh | 100.5 | 100.7 | 101 |

(b)

(a)


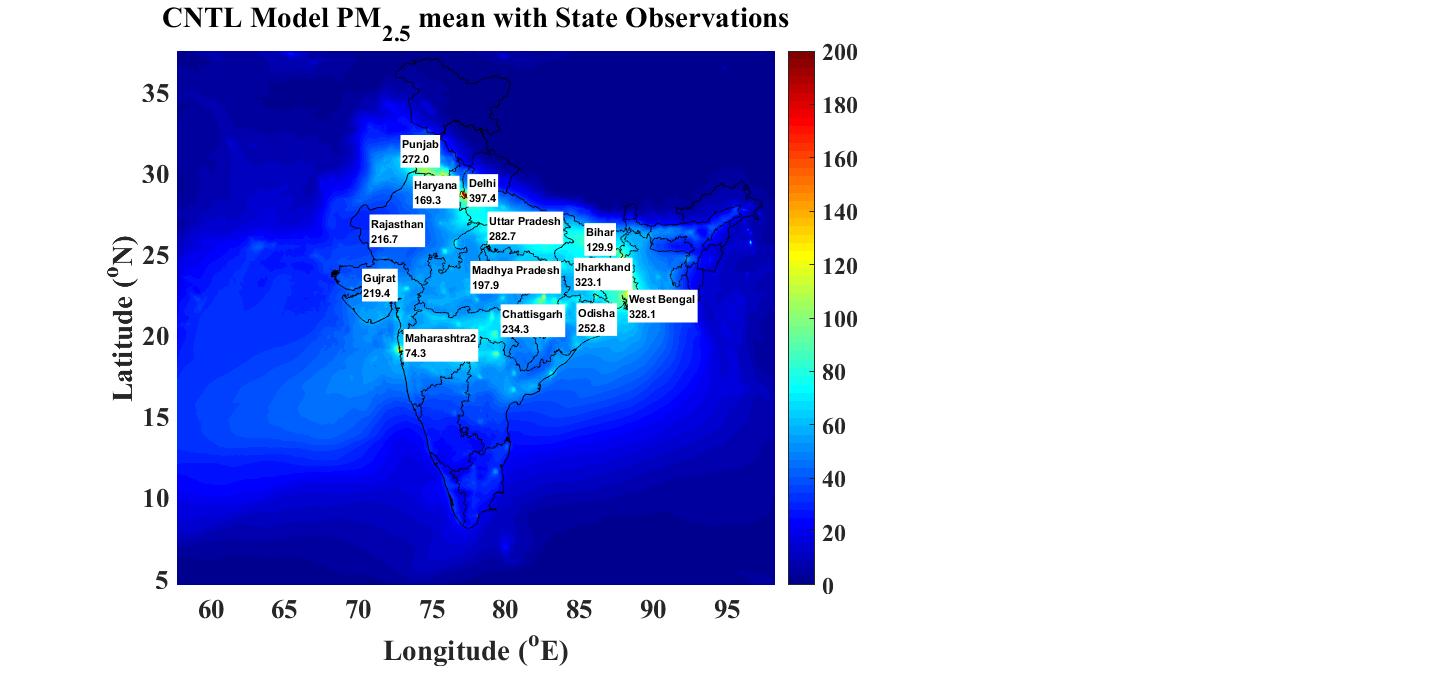

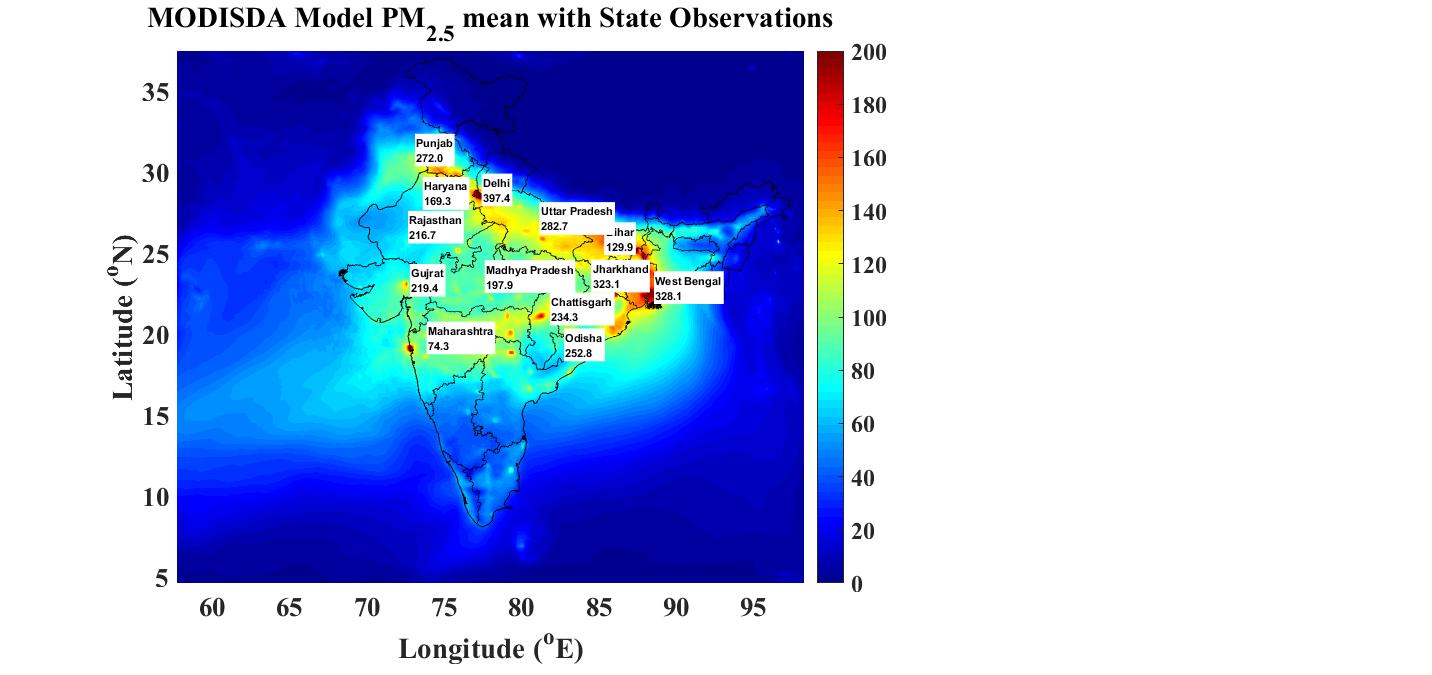

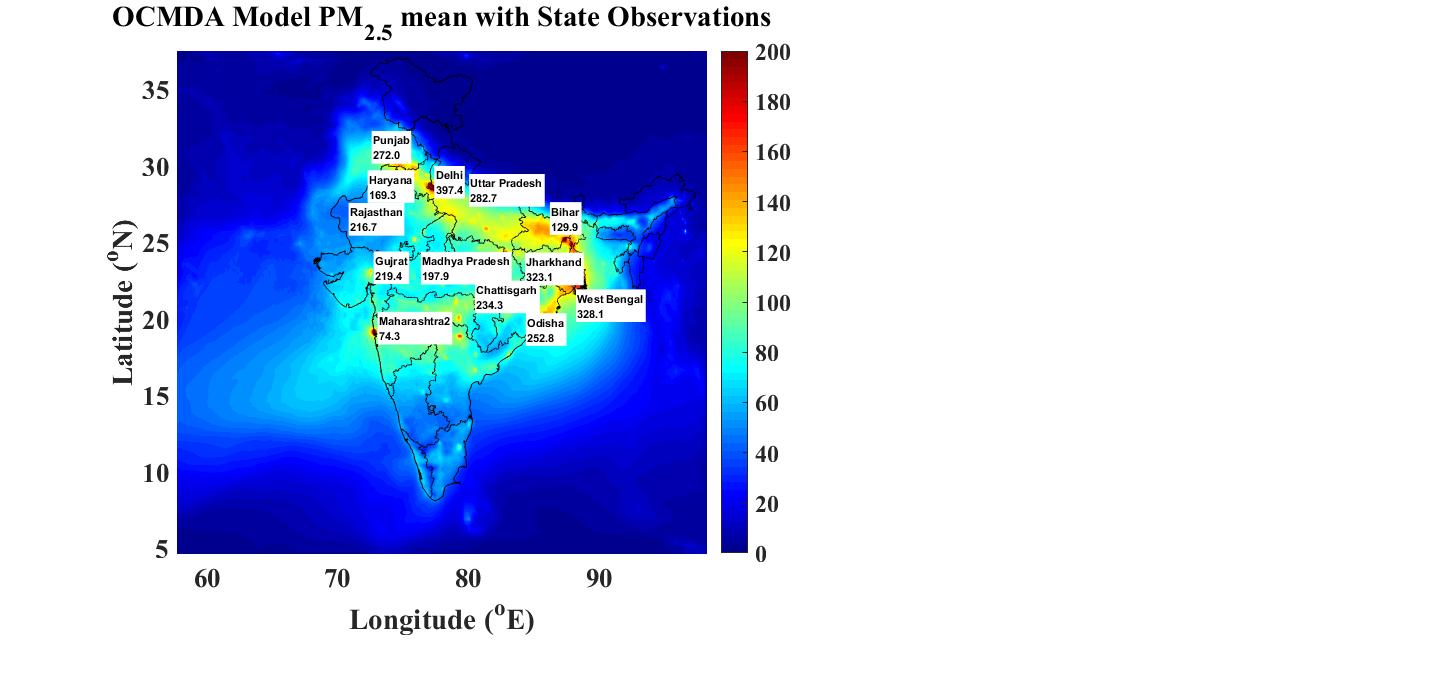


(c)

Figure S16. Spatial distribution of mean surface PM₂.₅ concentrations (µg m⁻³) simulated by (a) CNTL, (b) MODISDA, and (c) OCMDA experiments over India for the study period. Overlaid labels show the mean observed PM₂.₅ values (µg m⁻³) derived from ground/state-level datasets.

Table S5. Category-wise forecast skill scores — Accuracy (ACC, %), False Alarm Ratio (FAR, %), Probability of Detection (POD, %), and Critical Success Index (CSI, %) — for CNTL, MODISDA, and OCMDA experiments for Day-1 to Day-3 forecasts across selected states. The table highlights cases where OCMDA captured high-AQI episodes that were missed or underestimated by MODISDA, and vice versa. Entries marked as “NAN” indicate that no corresponding AQI event was observed in the model during that forecast period.

| **Forecast** | **CNTL_ACC** | **CNTL_FAR** | **CNTL_POD** | **CNTL_CSI** | **MODISDA_ACC** | **MODISDA_FAR** | **MODISDA_POD** | **MODISDA_CSI** | **OCMDA_ACC** | **OCMDA_FAR** | **OCMDA_POD** | **OCMDA_CSI** |
| --- | --- | --- | --- | --- | --- | --- | --- | --- | --- | --- | --- | --- |
| Delhi | | | | | | | | | | | | |
| Day1 | 100 | 0 | 100 | 100 | 100 | 0 | 100 | 100 | 100 | 0 | 100 | 100 |
| Day2 | 98 | 0 | 98 | 98 | 100 | 0 | 100 | 100 | 100 | 0 | 100 | 100 |
| Day3 | 89.568 | 0 | 89.138 | 89.138 | 100 | 0 | 100 | 100 | 100 | 0 | 100 | 100 |
|  |  |  |  |  |  |  |  |  |  |  |  |  |
| Gujarat | | | | | | | | | | | | |
| Day1 | 26.380 | 10 | 3.6290 | 3.6144 | 85.58 | 15.93 | 100 | 84.06 | 88.03 | 12.27 | 97.98 | 86.17 |
| Day2 | 36.754 | 0 | 15.111 | 15.111 | 78.14 | 22.68 | 100 | 77.31 | 82.11 | 19.35 | 100 | 80.64 |
| Day3 | 28.776 | 0 | 1.4925 | 1.4925 | 76.25 | 24.71 | 100 | 75.280 | 76.25 | 24.71 | 100 | 75.28 |
|  |  |  |  |  |  |  |  |  |  |  |  |  |
| Jharkhand | | | | | | | | | | | | |
| Day1 | 46.31 | 12.96 | 36.862 | 34.944 | 81.595 | 19.047 | 100 | 80.95 | 81.59 | 19.0476 | 100 | 80.952 |
| Day2 | 39.40 | 6.66 | 23.829 | 23.430 | 81.456 | 19.243 | 100 | 80.75 | 81.45 | 19.2439 | 100 | 80.756 |
| Day3 | 23.02 | 31.57 | 16.595 | 15.415 | 88.48 | 11.98 | 100 | 88.01 | 88.48 | 11.9850 | 100 | 88.014 |
|  |  |  |  |  |  |  |  |  |  |  |  |  |
| Maharashtra | | | | | | | | | | | | |
| Day1 | 80.981 | 100 | 0 | 0 | 11.9618 | 91.11 | 100 | 8.888 | 11.96 | 91.11 | 100 | 8.88 |
| Day2 | 59.189 | 100 | 0 | 0 | 21.256 | 93.156 | 100 | 6.612 | 25.15 | 94.12 | 100 | 7.891 |
| Day3 | 41.12 | NAN | 0 | 0 | 8.56 | 92.012 | 100 | 10.21 | 8.15 | 91.01 | 100 | 8.126 |
|  |  |  |  |  |  |  |  |  |  |  |  |  |
| Madhya Pradesh | | | | | | | | | | | | |
| Day1 | 75.460 | NAN | 0 | 0 | 27.91 | 74.603 | 100 | 25.39 | 53.98 | 65.3508 | 98.75 | 34.497 |
| Day2 | 74.503 | NAN | 0 | 0 | 32.450 | 73.09 | 96.10 | 26.61 | 47.35 | 68.4684 | 90.9090 | 30.56 |
| Day3 | 72.302 | NAN | 0 | 0 | 30.93 | 71.69 | 97.40 | 28.08 | 26.97 | 77.6315 | 66.2337 | 20.07 |
|  |  |  |  |  |  |  |  |  |  |  |  |  |
| Odisha | | | | | | | | | | | | |
| Day1 | 48.466 | 0 | 24.663 | 24.663 | 95.092 | 4.405 | 97.309 | 93.13 | 92.63 | 8.71 | 98.65 | 90.1639 |
| Day2 | 47.682 | 0 | 29.147 | 29.147 | 99.00 | 0 | 98.65 | 98.65 | 99.33 | 0 | 99.1031 | 99.1031 |
| Day3 | 22.442 | NAN | 0 | 0 | 95.37 | 5.622 | 100 | 94.37 | 95.37 | 5.622 | 100 | 94.377 |
|  |  |  |  |  |  |  |  |  |  |  |  |  |
| Punjab | | | | | | | | | | | | |
| Day1 | 19.325 | 0 | 1.8656 | 1.8656 | 50.613 | 0 | 39.92 | 39.925 | 48.773 | 0 | 37.686 | 37.686 |
| Day2 | 19.205 | NAN | 0 | 0 | 49.006 | 0 | 36.88 | 36.8 | 45.03 | 0 | 31.967 | 31.967 |
| Day3 | 22.661 | 0 | 2.2727 | 2.2727 | 52.158 | 0 | 39.54 | 39.54 | 50.359 | 0 | 37.272 | 37.272 |
|  |  |  |  |  |  |  |  |  |  |  |  |  |
| Rajasthan | | | | | | | | | | | | |
| Day1 | 34.969 | NAN | 0 | 0 | 49.69 | 26.470 | 35.37 | 31.380 | 34.969 | NAN | 0 | 0 |
| Day2 | 27.152 | NAN | 0 | 0 | 35.76 | 34.14 | 24.54 | 21.774 | 27.1523 | NAN | 0 | 0 |
| Day3 | 28.057 | NAN | 0 | 0 | 32.37 | 0 | 6 | 6 | 28.057 | NAN | 0 | 0 |
|  |  |  |  |  |  |  |  |  |  |  |  |  |
| Uttar Pradesh | | | | | | | | | | | | |
| Day1 | 46.01 | 0 | 35.055 | 35.055 | 86.503 | 13.968 | 100 | 86.03 | 86.503 | 13.96 | 100 | 86.0317 |
| Day2 | 52.31 | 8.8 | 46.153 | 44.186 | 85.430 | 15.120 | 100 | 84.879 | 85.430 | 15.120 | 100 | 84.879 |
| Day3 | 48.56 | 0 | 35.874 | 35.874 | 90.6474 | 10.44 | 100 | 89.558 | 91.36 | 9.387 | 99.55 | 90.243 |
|  |  |  |  |  |  |  |  |  |  |  |  |  |
| West Bengal | | | | | | | | | | | | |
| Day1 | 91.717 | 0 | 91.428 | 91.428 | 100 | 0 | 100 | 100 | 100 | 0 | 100 | 100 |
| Day2 | 100 | 0 | 100 | 100 | 100 | 0 | 100 | 100 | 100 | 0 | 100 | 100 |
| Day3 | 87.769 | 0 | 87.265 | 87.265 | 100 | 0 | 100 | 100 | 100 | 0 | 100 | 100 |
|  |  |  |  |  |  |  |  |  |  |  |  |  |
| Bihar | | | | | | | | | | | | |
| Day1 | 14.72 | 0 | 10.897 | 10.897 | 95.3987 | 0 | 95.1923 | 95.1923 | 95.3987 | 0 | 95.1923 | 95.192 |
| Day2 | 16.225 | 0 | 13.058 | 13.058 | 99.6688 | 0 | 99.6563 | 99.6563 | 99.006 | 0 | 98.9690 | 98.969 |
| Day3 | 15.827 | 0 | 12.359 | 12.359 | 100 | 0 | 100 | 100 | 100 | 0 | 100 | 100 |

**Refrences:**

- - - 1. Morrison, H., Thompson, G. & Tatarskii, V. Impact of cloud microphysics on the development of trailing stratiform precipitation in a simulated squall line: Comparison of one- and two-moment schemes. Monthly Weather Review 137, 991–1007 (2009). <https://doi.org/10.1175/2008MWR2556.1>
      2. Iacono, M. J., Delamere, J. S., Mlawer, E. J., Shephard, M. W., Clough, S. A. & Collins, W. D. Radiative forcing by long‐lived greenhouse gases: Calculations with the AER radiative transfer models. Journal of Geophysical Research: Atmospheres 113, D13103 (2008). <https://doi.org/10.1029/2008JD009944>
      3. Grell, G. A. & Freitas, S. R. A scale and aerosol aware stochastic convective parameterization for weather and air quality modeling. Atmospheric Chemistry and Physics 14, 5233–5250 (2014). <https://doi.org/10.5194/acp-14-5233-2014>
      4. Tewari, M. et al. Implementation and verification of the unified Noah land surface model in the WRF model. 20th Conference on Weather Analysis and Forecasting/16th Conference on Numerical Weather Prediction (2004). [Available online: <https://opensky.ucar.edu/islandora/object/conference%3AWWRF2004Tewari>]
      5. Nakanishi, M. & Niino, H. An improved Mellor–Yamada Level-3 model: Its numerical stability and application to a regional prediction of advection fog. Boundary-Layer Meteorology 119, 397–407 (2006). <https://doi.org/10.1007/s10546-005-9030-8>
      6. Janjić, Z. I. The surface layer in the NCEP Eta Model. 11th Conference on Numerical Weather Prediction, American Meteorological Society, Norfolk, VA (1996). [Available at AMS archives]
      7. Chin, M. et al. Atmospheric sulfur cycle simulated in the global model GOCART: Model description and global properties. Journal of Geophysical Research: Atmospheres 105, 24671–24687 (2000). <https://doi.org/10.1029/2000JD900384>
      8. Ginoux, P., Chin, M., Tegen, I., Prospero, J. M., Holben, B., Dubovik, O. & Lin, S. J. Sources and distributions of dust aerosols simulated with the GOCART model. Journal of Geophysical Research: Atmospheres 106, 20255–20273 (2001). <https://doi.org/10.1029/2000JD000053>
      9. Janssens-Maenhout, G. et al. EDGAR-HTAP: A harmonized gridded air pollution emission dataset based on national inventories. Earth System Science Data 7, 449–498 (2015). <https://doi.org/10.5194/essd-7-449-2015>
      10. Venkataraman, C. et al. Source influence on emission pathways and ambient PM2.5 pollution over India (2015–2050). Atmospheric Chemistry and Physics 18, 8017–8039 (2018). <https://doi.org/10.5194/acp-18-8017-2018>
      11. SAFAR-High Resolution Emission Inventory of Megacity Delhi 2018 MoES, Govt. of India (2018) (Special Scientific Report; SAFAR-Delhi-2018-A, ISSN: 0252-1075) <http://safar.tropmet.res.in/source.pdf>
      12. Guenther, A. B., Karl, T., Harley, P., Wiedinmyer, C., Palmer, P. I. & Geron, C. Estimates of global terrestrial isoprene emissions using MEGAN (Model of Emissions of Gases and Aerosols from Nature). Atmospheric Chemistry and Physics 6, 3181–3210 (2006). <https://doi.org/10.5194/acp-6-3181-2006>
      13. Wiedinmyer, C. et al. The Fire INventory from NCAR (FINN): A high resolution global model to estimate the emissions from open burning. Geoscientific Model Development 4, 625–641 (2011). <https://doi.org/10.5194/gmd-4-625-2011>
      14. Govardhan, G., S. K. Satheesh, K. K. Moorthy, and R. Nanjundiah, 2019: Simulations of black carbon over the Indian region: Improvements and implications of diurnality in emissions. Atmos. Chem. Phys., 19, 8229–8241, https://doi.org/10.5194/acp-19-8229-2019.
